# Examining Schizotypal Personality Scales Within and Across Interpersonal Circumplex Surfaces

**DOI:** 10.1177/10731911221143354

**Published:** 2023-01-11

**Authors:** A. Esin Asan, Aaron L. Pincus

**Affiliations:** 1 The Pennsylvania State University, University Park, USA

**Keywords:** personality assessment, schizotypal personality disorder, interpersonal circumplex, interpersonal construct validation, bootstrapping

## Abstract

Differing perspectives on the operationalization of schizotypal personality pathology (STPP) have led to numerous multidimensional assessment measures. The current study applied the interpersonal construct validation approach to self-report data from 856 undergraduate students to formally examine the interpersonal content, similarities, and differences in the subscales of four measures of STPP within and across two interpersonal circumplex surfaces using a bootstrapping methodology for computing confidence intervals around circumplex structural summary method parameters. Results suggested that negative-like expressions of STPP are prototypically and distinctively interpersonal constructs associated with cold and socially avoidant interpersonal problems and hypersensitivity to others’ warmth and affection. Positive-like expressions of STPP as assessed by two out of four measures were prototypically and distinctively interpersonal constructs associated with vindictiveness. Across four measures, there was notable overlap in interpersonal correlates among related subscales, suggesting convergent validity. However, subscales containing social anxiety content were associated with more submissive (i.e., socially avoidant) interpersonal problems than subscales without social anxiety content.

Schizotypy was seminally defined by Paul Meehl as a personality organization contributing to a latent liability for schizophrenia, which can manifest phenotypically in behavior, cognition, and neurodevelopment (Meehl, 1962, 1990). This definition assumed that there were individuals (i.e., *schizotypes*) who displayed transient signs of schizophrenia-like cognitive and behavioral symptoms as well as social impairment and interpersonal vulnerability (e.g., subtle thought disorder or excessive interpersonal fear). Consistent with this theory, early measures of schizotypy, such as Chapmans’s “proneness to psychosis” scales (Chapman et al., 1978; Eckblad & Chapman, 1983), assessed schizotypy as one of the indicators of liability for schizophrenia, in addition to laboratory measures, psychometric indexes, and family history of schizophrenia (Lenzenweger, 2010).

Current multidimensional models use the conceptual framework of schizotypy to encompass a broad spectrum of psychopathology, including schizotypal and schizoid personality disorders, the schizophrenia prodrome, and subclinical expressions (Kwapil & Barrantes-Vidal, 2015). These contemporary conceptualizations of schizotypy, reflecting odd, eccentric, and disorganized cognitive, affective, and behavioral processes and their associated impacts on self and relational functioning, offer a broad unifying construct for understanding the etiology, development, and expression of schizophrenia-spectrum psychopathology. However, a lack of consensus across models has led to disagreement regarding content and number of dimensions, poorly integrated research literature, and multiple different assessment measures. This has been exacerbated by imprecise use of the term schizotypy when research actually assesses its specific and distinct expressions such as schizotypal personality disorder (STPD; Kwapil & Barrantes-Vidal, 2015). The current study focuses on the assessment of STPD and schizotypal traits (referred to here as schizotypal personality pathology—STPP) as characterological expressions of schizotypy. Specifically, we compared four alternative assessment instruments by employing methods of interpersonal construct validation (Wiggins & Broughton, 1991; Zimmermann & Wright, 2017).

STPP can be conceptualized as a prominent nonpsychotic personality expression of schizotypy rather than a liability for psychosis. At a high level of clinical severity, STPP may be formally diagnosed as STPD. At moderate levels, STPP may present as a set of broad and stable features and is associated with variation in social functioning (Fonseca-Pedrero et al., 2014; Lenzenweger, 2011). While the *Diagnostic and Statistical Manual of Mental Disorders* (5th ed.; *DSM-5*; American Psychiatric Association [APA], 2013) includes STPD in its Personality Disorders chapter, it is also referenced in the Schizophrenia-Spectrum Disorders chapter. Notably, past research finds that while schizotypy is genetically and developmentally related to schizophrenia, STPD exhibits higher rates of comorbidity with other personality disorders than with schizophrenia-related disorders (Raine, 2006).

## Measures of Schizotypy and Schizotypal Personality Pathology

### The Multidimensional Schizotypy Scale

One of the most widely accepted models of schizotypy indicates positive schizotypy and negative schizotypy as distinct lower level factors (Kwapil & Barrantes-Vidal, 2015; Vollema & van den Bosch, 1995). This model has been replicated across samples and cultures (Chan et al., 2015; Kwapil et al., 2012; Stefanis et al., 2002). The positive dimension includes perceptual disturbances, disruptions in thought content, and paranoia. The negative dimension includes flattened affect, social disinterest, anhedonia, and avolition. Expanding on the two-factor model, Kwapil, Gross, Silvia and colleagues (2018) later demonstrated the construct validity of an additional factor of disorganized schizotypy, which includes confusion, racing thoughts, disrupted speech, and loose associations. The Multidimensional Schizotypy Scale (MSS; Kwapil, Gross, Burgin, et al., 2018; Kwapil, Gross, Silvia, et al., 2018) is a measure for assessing positive, negative, and disorganized schizotypy. The MSS was developed by using the current multidimensional models of schizotypy as a unifying construct for expressions of schizophrenia-spectrum psychopathology, including STPP. Therefore, for the purposes of this study as a construct validation of STPP measures, we will refer to the MSS as a measure of STPP. Recent studies have shown that negative and positive schizotypy assessed using the MSS is robustly associated with STPD symptomatology (Kemp et al., 2021; Kwapil et al., 2022). However, this overlap has not yet been examined through the lens of interpersonal construct validation (Wiggins & Broughton, 1991; Zimmermann & Wright, 2017).

### The Schizotypal Personality Questionnaire

Following the *Diagnostic and Statistical Manual of Mental Disorders* (3rd ed., rev.; *DSM-III-R*; APA, 1987) symptom criteria of STPD, Raine and colleagues (1994) constructed a three-factor measure, the Schizotypal Personality Questionnaire (SPQ; Raine, 1991), with cognitive-perceptual, interpersonal, and disorganized subscales, which has since been replicated multiple times (Fossati et al., 2003; Reynolds et al., 2000). In this operationalization of STPD, the cognitive-perceptual abnormalities are expressions of positive schizotypy (ideas of reference, magical thinking, unusual perceptual experiences, and suspiciousness). Some of the interpersonal features (namely, no close friends, constricted affect, and social anhedonia) are expressions of negative schizotypy; however, other interpersonal features such as social anxiety and neuroticism are not typically considered to align with negative schizotypy. Odd behavior and odd speech are included under the disorganized subscale. Although the SPQ was constructed based on the nine symptoms of *DSM-III-R* schizotypal personality disorder, it is widely used as a multidimensional measure of schizotypy rather than a diagnostic scale (Chmielewski & Watson, 2008). However, given the subscales of the SPQ emerged from a post hoc factor analysis of items based on *DSM-III-R* schizotypal personality disorder symptoms, the measure provides limited coverage of current multidimensional models of schizotypy despite the overlap in content. The SPQ and its Brief Revised version (SPQ-BR; Cohen et al., 2010) developed and validated subscales that measure Raine and colleagues (1994) three domains of STPD. The updated version of SPQ-BR (Davidson et al., 2016) proposed a four-factor structure with a better fit, introducing social anxiety as a fourth higher-order factor correlating with but not loading on to the interpersonal factor. This social anxiety subscale includes items from the interpersonal scale of SPQ-BR that measure discomfort and nervousness in social situations that involve unfamiliar people.

### The Five-Factor Schizotypal Inventory

Over the past two decades, a large empirical literature has accrued to support the use of the Five-Factor Model (FFM) of personality traits to conceptualize personality disorders (J. D. Miller & Widiger, 2020; Widiger & Costa, 2013). The Five-Factor Schizotypal Inventory (FFSI; Edmundson et al., 2011; Moorman & Samuel, 2018) was developed using the FFM as a framework to create a measure of schizotypal personality traits. The FFSI includes nine subscales that assess maladaptive trait facets across four FFM domains. Specifically, these subscales assess interpersonal suspiciousness (reflecting low Agreeableness); social anhedonia, social isolation and physical anhedonia (reflecting low Extraversion); social anxiousness and social discomfort (reflecting high Neuroticism); and aberrant perceptions, oddness and eccentricity, and aberrant ideas (reflecting maladaptively high Openness). The FFSI overlaps with the SPQ by operationalizing high Neuroticism (social anxiety and discomfort) as a dimension of STPP using items that assess nervousness and discomfort when around other people.

### The Personality Inventory for *DSM*-5 Alternative Model for Personality Disorders

The Alternative Model for Personality Disorders (AMPD) posits the domains of detachment and psychoticism and their facets (Suspiciousness, Restricted Affectivity and Withdrawal; Unusual Beliefs & Experiences, Eccentricity, and Perceptual Dysregulation) as Criterion B traits of STPD in the Section III of *DSM-5* (APA, 2013). These two domains have been conceptualized and replicated as maladaptive variants of FFM Extraversion and Openness respectively (see Widiger & McCabe, 2020 for a review). The Personality Inventory for *DSM-5* (PID-5; Krueger et al., 2012) provides the official assessment of the *DSM-5* dimensional trait model and includes subscales that measure the six facets listed as Criterion B traits of STPD. Previous research on the association of PID-5 domains with multidimensional schizotypy assessed by the MSS has shown large associations between positive schizotypy and the PID-5 psychoticism domain and between negative schizotypy and the PID-5 detachment domain (Kemp et al., 2022). In the same study, PID-5 domains accounted for approximately half of the variance in each of the schizotypy dimensions. These findings provide additional support for the use of these two PID-5 domains as a measure of STPP.

### Convergences and Divergences

Although these four measures all assess STPP multidimensionally through subscales, there are distinctions in their conceptualization of STPP. Both the FFSI and the PID-5 are measures developed using expressions of the maladaptive variants of five-factor personality traits and are therefore not based on symptom criteria like the SPQ or based on the multidimensional models of schizotypy like the MSS. Overall, the MSS and SPQ assess STPP as an expression of schizophrenia-spectrum pathology, whereas the FFSI and PID-5 are trait frameworks that characterize STPP using pathological variants of general personality traits. Theoretically, the MSS significantly diverges from the other three measures, as it was developed using current multidimensional models of schizotypy. Therefore, caution should be used in considering the SPQ, FFSI, and PID-5 as a measure of schizotypy. The symptoms assessed by the SPQ and the traits assessed by the FFSI and the PID-5 can be considered specific expressions of schizotypy but are not synonymous with current multidimensional models of schizotypy. However, there may be an overlap in the dimensions of STPP assessed by each measure.

In terms of the convergences and divergences in content and dimensions, research generally supports that schizotypal traits include unusual beliefs and perceptions (a dimension of positive symptom-like experiences) and social, interpersonal, and affective difficulties (a dimension of negative symptom-like experiences). All measures include these two dimensions as subscales. Notably, odd or eccentric speech and behavior have either been included under a disorganized factor, as exemplified by the three-factor structure of the symptom-based models (Kwapil, Gross, Silvia, et al., 2018; Raine, 1994), or subsumed by the Psychoticism/Openness dimension as exemplified by the personality-based models (APA, 2013; Edmundson et al., 2011).

In addition, the SPQ and the FFSI are distinctive from the MSS and the PID-5 because the former pair of instruments includes content related to social anxiety within their operationalization of STPP. The SPQ Interpersonal subscale includes social anhedonia and withdrawal, as well as nervousness, guardedness, and interpersonal discomfort (Gross et al., 2018), and the FFSI Neuroticism domain includes social anxiousness and discomfort. The PID-5 diverges from the SPQ and the FFSI as well, as PID-5 facets that assess anxiety are not included as primary Criterion B traits of STPD, and therefore social disinterest is emphasized over social discomfort. These content differences may also influence the interpersonal characteristics of what each instrument and its subscales assess.

Given multiple measures assessing STPP, identifying the points of interpersonal convergence and divergence among measures would provide evidence for construct validity. As Moorman and Samuel (2018) note, a limitation of the studies that have investigated the convergent validity between multiple measures of schizotypal thinking and perception has been their focus on observed correlations between measures. There is still a need for an additional examination of the overlap and distinctiveness between measures of schizotypal thinking and perception over and above assessing the strength of observed correlations between different scales and subscales. Previously, Suzuki et al. (2017) evaluated nomological network similarities using associations with external variables related to psychoticism, such as social adjustment and dating frequency. Another useful method is to examine the convergences and divergences of the interpersonal correlates of different STPP measures.

## The Interpersonal Paradigm and the Interpersonal Circumplex

Schizotypy and STPP are generally associated with pervasive disinterest, discomfort, and dysfunction in interpersonal relationships as well as variation in perceptions and representations of self and other. For example, previous research has broadly linked STPP to social withdrawal and flat affect (Tyrka et al., 1995), nonverbal interpersonal sensitivity (A. B. Miller & Lenzenweger, 2014), and self-referential hypermentalization (Wastler & Lenzenweger, 2019). More specifically, negative schizotypy is associated with diminished social contact (Kwapil et al., 2020), diminished interest in friendships and dating (Kemp et al., 2021), and positive schizotypy more generally. As suspiciousness and paranoia are core interpersonal components of positive schizotypy due to beliefs that others are threatening and malevolent (Horton et al., 2014), interpersonal functioning provides a useful lens through which to examine and integrate the various measures of STPP.

**Figure 1.**
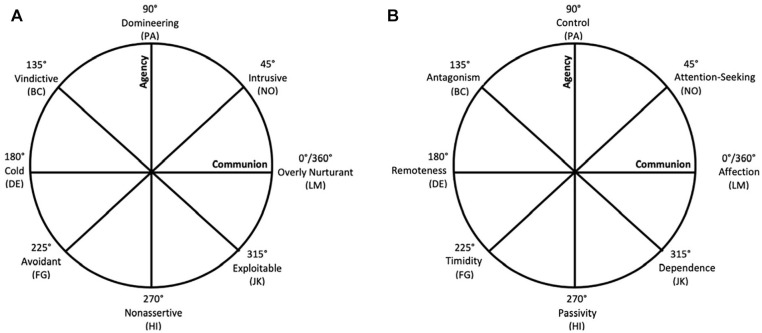
The Interpersonal Problems (A) and Sensitivities (B) Circumplexes.

The interpersonal paradigm of personality assessment (Wiggins, 2003) treats interpersonal relationships and experiences as integral in all domains of functioning and presents a framework that provides an integrative nexus for the study of personality and psychopathology (Hopwood et al., 2019, 2021; Leary, 1957; Pincus et al., 2020; Pincus & Hopwood, 2012; Wright et al., 2021). Specific to this current study, interpersonal construct validation (Wiggins & Broughton, 1985, 1991; Zimmermann & Wright, 2017) clarifies the meaning of related external constructs by locating them within the two-dimensional space defined by the Interpersonal Circumplex (IPC; Fournier et al., 2011; Figure 1). Capitalizing on the geometric properties of IPC’s circular structure, this framework establishes interpersonal construct validity by quantitatively defining the characteristics of an interpersonal surface and thus the location of the external construct within the surface. The IPC assumes that two basic themes define social and interpersonal relationships: dominance/agency (y-axis), related to autonomy, mastery, and superiority, and affiliation/communion (x-axis), related to helping and forming relationships with others (Bakan, 1966). When these two dimensions are placed as axes in a two-dimensional space and the various combinations of their interpersonal content are plotted, a circular pattern referred to as a *circumplex* emerges (Wiggins, 1979).

There are a variety of circumplex surfaces representing different interpersonal constructs, including interpersonal problems (Alden et al., 1990; Soldz et al., 1995) and sensitivities (Hopwood et al., 2011). Differences in content lead these measures to have distinct interpersonal implications, but both divide the IPC into eight octants, with midpoints of 45°, 90°, 135°, 180°, 225°, 270°, 315°, and 360°. The octants are identified with a two-letter label to mark locations across different surfaces, which represent central themes of the octants reflecting eight distinct blends of dominance/agency and affiliation/communion. IPC measures dictate that the relationship between octants is inversely proportional to the angular distance between the octants (Gurtman & Pincus, 2003). Meaning, scales that are adjacent have conceptually similar content and are positively correlated. Scales that are diametrically opposed to each other are conceptual opposites and strongly negatively correlated. Scales that are orthogonal to each another are conceptually unrelated and correlated near zero.

**Figure 2.**
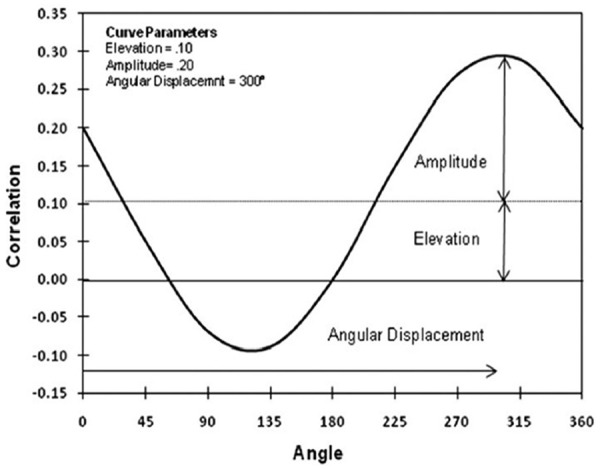
Illustration of the Cosine Curve Parameters Associated With the Structural Summary Method for Circumplex Data (Trucco et al., 2013).

Due to the circular structure of the IPC, if an external construct (i.e., Detachment) has prototypical and distinctive interpersonal content, the correlations between scores on the construct and each IPC octant score will form a cosine curve when plotted (Gurtman, 1994). The structural summary method for circumplex data (SSM; Gurtman, 1992; Gurtman & Pincus, 2003; Wright et al., 2009) uses the characteristics of this cosine curve, referred to as an interpersonal profile (Figure 2), to quantify the strength and distinctiveness of the interpersonal content, the dominant interpersonal theme and interpersonal prototypicality of the external construct using the four parameters elevation, amplitude, and angular displacement, and prototypicality (*R*^2^), respectively. Elevation (*e*) refers to the association between the criterion variable and the general factor of the IPC measure. Amplitude (*a*) refers to profile differentiation (i.e., the difference between the average correlation and the peak correlation of the profile)—to what extent the external construct exhibits distinctive interpersonal content. Angular displacement (*Θ*) characterizes the main interpersonal theme of the external construct and refers to the angular distance from 0° to the peak correlation of the profile. *R*^2^ indicates the degree to which a perfect cosine curve fits the profile, representing prototypicality (vs. complexity) of the profile. An *R*^2^ value of .80 indicates a good fit to a cosine curve and a value of .70 indicates an acceptable fit (Zimmermann & Wright, 2017). Although the interpretation of the elevation parameter does not depend on prototypicality, the amplitude and angular displacement parameter values are only interpretable if the profile displays at least acceptable prototypicality. The SSM additionally provides vector scores for dominance and affiliation, which are computed using trigonometrically weighted combinations of the correlations between the external construct and the octant scores (Gurtman & Pincus, 2003). A bootstrapping methodology for the SSM (Zimmermann & Wright, 2017) computes confidence intervals (CIs) around SSM parameters to allow researchers to test inferential questions about convergent and discriminant validity by determining whether the interpersonal profile parameters of two external constructs are statistically distinct from each other. Recent research has employed this method to examine the interpersonal construct validity of numerous personality and personality disorder constructs, including the FFM and its facets (Du et al., 2021), Criterion A of the AMPD (Dowgwillo et al., 2018), the dark triad (Dowgwillo & Pincus, 2017), and rejection sensitivity (Cain et al., 2017).

### Interpersonal Analyses of Schizotypal Personality Pathology

Early research failed to demonstrate strong and specific associations between STPD and IPC (Pincus & Wiggins, 1990; Soldz et al., 1993). However, more recent research has consistently found moderate-to-large associations between features of STPD and interpersonal coldness, vindictiveness, and social avoidance (Williams & Simms, 2016; Wright et al., 2012). Notably, a recent meta-analytic review of interpersonal dysfunction in personality disorders found that STPD had a prototypical and distinctive IPC profile reflecting a cold/distant interpersonal style (*Θ* = 171.31°; Wilson et al., 2017). These inconsistent findings may be due to the multidimensionality of schizotypal pathology, as different schizotypy dimensions are associated with distinct patterns of interpersonal impairment. Notably, Wright and colleagues (2012) suggested that due to its multidimensionality as an expression of schizotypy, STPD appears to be comprised of both specific and non-specific interpersonal content. For example, AMPD Criterion B psychoticism traits are generally interpersonally undifferentiated, only modestly associated with hostility and vindictiveness as a result of finding oneself at odds with and suspicious toward others due to discrepant realities. However, AMPD Criterion B detachment traits specifically capture interpersonal separation and mistrust, exhibiting strong associations with coldness and disaffiliation.

## Current Study

No prior studies have formally examined the overlap and distinctiveness among the abovementioned measures within the interpersonal construct validation framework. This study uses the IPC to identify the various interpersonal characteristics of STPP assessed by four widely used measures. To establish convergent and discriminant validity, we employ a bootstrapping procedure to compute CI for SSM parameters (Zimmermann & Wright, 2017) to test the interpersonal convergences and divergences of STPP subscales and provide evidence for interpersonal construct validity (Gurtman, 1992, 2009; Wiggins & Broughton, 1985, 1991; Zimmermann & Wright, 2017). To date, the focus of research on the interpersonal nature of STPD has been limited to interpersonal traits and interpersonal problems. To provide a richer interpersonal construct validation, the current study employs an additional IPC surface assessed with the Interpersonal Sensitivities Circumplex (ISC; Hopwood et al., 2011). The main objective of this study is to evaluate the interpersonal construct validity of four measures of STPP by empirically identifying convergences and divergences among their associations with multiple IPC measures and empirically testing their SSM parameter differences. A secondary aim of this study is to explore whether the inclusion of social anxiety in the content of two of the four measures has an impact on scale convergences and divergences. Notably, as recent studies have demonstrated that the variance accounted for by total scores of schizotypy scales is on average only half of the variance accounted for by the subscales (Kemp et al., 2021), we opted to focus on subscales over total scores for our analyses. Analyses using total scores on the four measures are presented in the supplementary material.

Although there are no consensus guidelines for interpreting SSM elevation and amplitude, heuristic cut-offs ranging from ≥.|10| to ≥.|15| have been proposed as constituting an elevated and/or differentiated profile (Dowgwillo et al., 2018; Dowgwillo & Pincus, 2017; Du et al., 2021; Grove et al., 2019; Williams & Simms, 2016; Wright et al., 2012; Wu et al., 2015). Due to the preliminary nature of the study and the use of a normative student sample which likely limited the range of STPP and interpersonal problems scores, we adopted the more liberal ≥.|10| cut-off for interpreting elevation and amplitude. The cut-off for prototypicality was *R*^2^ = .70 (acceptable fit; Zimmermann & Wright, 2017).

Based on previous research and theory, we made the following predictions:

**Hypothesis 1 (H1):** STPP assessed by all subscales will be positively associated with general interpersonal distress (Inventory of Interpersonal Problems Short Circumplex [IIP-SC] *e* ≥ .10).
**Hypothesis 2 (H2):** Angular displacement (*Θ*) of interpersonal sensitivity profiles of all subscales will be interpersonally opposite to the interpersonal problems profiles (see Hopwood et al., 2011). For the current study, this reflects sensitivity to affection (LM).
**Hypothesis 3 (H3):** With regard to angular displacements (*Θ*), subscales assessing anhedonia and isolation/withdrawal will be associated with cold (DE) or cold-submissive (FG) interpersonal themes, and subscales measuring suspiciousness, ideas of reference, and paranoid ideation (i.e., positive/cognitive perceptual features) will show cold-dominant (BC) interpersonal themes.
**Hypothesis 4 (H4):** Subscales assessing suspiciousness and ideas of reference will display positive associations with general interpersonal hypersensitivity (ISC e ≥ .10).
**Hypothesis 5 (H5):** Based on content, the following sets of sub-scales will have statistically convergent angular displacement (*Θ*) parameters within each surface:
  (a) subscales assessing social disinterest and anhedonia, i.e., MSS Negative Schizotypy, FFSI Low Extraversion and PID-5 detachment subscales,
  (b) subscales assessing social anxiety i.e., SPQ Interpersonal and FFSI High Neuroticism subscales,
  (c) subscales assessing positive and cognitive-perceptual schizotypal personality features, i.e., SPQ Cognitive-Perceptual and MSS Positive Schizotypy, FFSI Low Agreeableness, FFSI High Openness and PID-5 psychoticism subscales.

## Method

We report how we determined our sample size, all data exclusions, all manipulations, and all measures in the study. Participants did not provide any other data than what is reported in this manuscript.

### Participants

Participants were 929 undergraduate students at a large northeastern university. The participants were recruited through a psychology department subject pool for course credit. Sample size was decided based on the recommendations proposed by Zimmermann and Wright (2017) to target for *n* = 500 and aim to have a sufficient sample of males and females for gender invariance analyses in a future study. A total of 31 participants generated incomplete protocols and were excluded based on the criterion of leaving more than 2% of the items blank. A total of 39 participants were excluded due to invalid protocols based on the criterion that their scores on the Personality Assessment Inventory Infrequency scale was more than 2.5 *SD*s above the mean in a normative sample (Morey, 1991). Three participants were excluded due to the observed detection of an invalid protocol that was not caught by the PAI Infrequency Scale. Overall, 856 complete and valid protocols were retained for analysis. The retained participants ranged in age from 18 to 65 (*M* = 19.11, *SD* = 3.07; see Table 1 for frequencies of other demographic variables).

**Table 1.** Sample Demographics.

Demographics	Frequency (%)
Sex	
Male	47.31
Female	52.34
Transgender Male	0.35
Transgender Female	0
Other	0
Sexual Orientation	
Heterosexual	89.32
Homosexual	3.40
Bisexual	5.52
Other	1.76
Race/Ethnicity	
African American	3.40
Asian	11.01
American Indian or Alaskan Native	0
Caucasian	69.32
Hispanic or Latino	3.63
Middle Eastern or North African	0.23
Native Hawaiian or Pacific Islander	1.05
Other	1.05
Biracial/Multiracial	10.30
Marital Status	
Single	71.11
Dating	27.49
Married	1.17
Divorced	0.23
English Primary Language	
Yes	93.57
No	6.43

### Schizotypal Personality Pathology Measures

Descriptive statistics, including internal consistencies (McDonald’s ω_t_), for all the MSS, SPQ, FFSI and PID-5 subscales are reported in Table 2.

**Table 2:** Descriptive Statistics for Schizotypal Personality Pathology Subscales.

Scale	*N*	*M*	*SD*	Minimum	Maximum	ω_*t*_
MSS Negative	831	3.26	3.99	0.00	24.00	0.86
MSS Positive	829	3.27	4.17	0.00	26.00	0.88
MSS Disorganized	830	3.66	5.42	0.00	25.00	0.94
SPQ Interpersonal	856	2.58	0.83	1.00	4.80	0.87
SPQ Cognitive-Perceptual	855	2.22	0.67	1.00	4.79	0.86
SPQ Disorganized	856	2.78	0.85	1.00	5.00	0.85
FFSI Agreeableness	855	2.43	0.74	1.00	4.70	0.56
FFSI Extraversion	855	2.06	0.68	1.00	4.33	0.95
FFSI Neuroticism	855	2.76	0.82	1.00	4.65	0.95
FFSI Openness	854	2.28	0.78	1.00	4.77	0.96
PID-5 Detachment	802	0.73	0.48	0.00	2.43	0.91
PID-5 Psychoticism	796	0.45	0.48	0.00	2.75	0.96

*Note.* Total *N* = 856; MSS = Multidimensional Schizotypy Scale; SPQ = Schizotypal Personality Questionnaire; FFSI = Five-Factor Schizotypal Inventory; PID-5 = Personality Inventory for *DSM*-5.

#### Multidimensional Schizotypy Scale

The MSS (Kwapil, Gross, Silvia, et al., 2018) is a 77-item true/false scale with subscales for negative, positive, and disorganized schizotypy symptoms. MSS was developed to assess non-psychotic schizotypy (including social anhedonia and detachment symptoms) that is characteristic of people with schizotypal personality disorder and schizophrenia prodrome. Although this measure was recently developed, robust initial research has established the validity and reliability of MSS and its brief form (Kemp et al., 2021; Kwapil, Gross, Burgin, et al., 2018).

#### Schizotypal Personality Questionnaire—Brief Revised Updated

The Schizotypal Personality Questionnaire-Brief Revised (SPQ-BRU; Davidson et al., 2016) is the updated version of the Schizotypal Personality Questionnaire-Brief Revised (SPQ-BR) developed by Cohen and colleagues (2010) as the brief version of Raine’s (1991) SPQ scale. The SPQ-BRU is a 32-item self-report scale on a 5-point Likert-type-scale (ranging from “strongly disagree” to “strongly agree”) and has three subscales, including Interpersonal, Cognitive-Perceptual, and Disorganized symptoms. The SPQ-BRU kept the item content but changed the item wording of SPQ-BR minimally such that all prompts were phrased in the first-person (“I. . .”) and none are phrased in the second-person (“You. . .”). In addition, SPQ-BRU proposed an alternative four subscale structure, as well, with a Social Anxiety subscale (SPQ-BRU-SA) that relates do but does not load onto the Interpersonal subscale.

#### Five-Factor Schizotypal Inventory

The Five-Factor Schizotypal Inventory (FFSI; Edmundson et al., 2011) is one of the Five-Factor Model Personality Disorder (FFMPD) self-report measures and includes 10-item subscales for nine maladaptive variants of FFM trait facets, corresponding to low Agreeableness (Interpersonal Suspiciousness), low Extraversion (Social Anhedonia, Social Isolation and Withdrawal, and Physical Anhedonia), high Neuroticism (Social Anxiousness and Social Discomfort), and high Openness (Aberrant Perceptions, Odd and Eccentric, and Aberrant Ideas). The items are rated on a 5-point Likert-type scale (ranging from “strongly disagree” to “strongly agree”). Initial validation of the FFSI demonstrated incremental validity beyond the other measures of STPP such as the SPQ (Edmundson et al., 2011), and additional research established its convergent and discriminant validity with other measures of the FFM and schizotypal thinking and perception (Crego & Widiger, 2016; Moorman & Samuel, 2018).

#### Personality Inventory for DSM-5

The PID-5 (Krueger et al., 2012) is a 220-item questionnaire designed to assess the *DSM-5* Section III dimensional trait model using a 4-point Likert-type scale (ranging from “very often false” to “often true”) for participants to describe how well a statement describes them. The 25 facet scales of the PID-5 can be organized into five domains of Negative Affectivity, Antagonism, Detachment, Disinhibition, and Psychoticism. Three facets of detachment (Suspiciousness, Restricted Affectivity, and Withdrawal) and psychoticism (Unusual Beliefs & Experiences, Eccentricity, and Perceptual Dysregulation) are used as diagnostic criteria for STPD in AMPD. For the purposes of this study, only the 57 items assessing the *DSM-5* AMPD STPD facets of Detachment and Psychoticism were used to calculate the total score (see supplemental material).

### Interpersonal Circumplex Measures

#### Inventory of Interpersonal Problems Short Circumplex

The IIP-SC (Soldz et al., 1995) is a 32-item measure that maps interpersonal problems (distressing behavioral excesses and inhibitions) onto the eight octants of the IPC. Items are rated on a 5-point Likert-type scale ranging from 0 (*not at all distressing*) to 4 (*extremely distressing*). In the current study, internal consistencies of IIP-SC octants were measured using McDonald’s ω_*t*_ and ranged from .60 (BC) to .87 (FG) with a mean ω_*t*_ of .77. Following the guidelines of Zimmermann and Wright (2017), a randomization test of hypothesized order relations (RANDALL; Tracey, 1997) was employed to ensure that the IIP–SC adhered to a circumplex structure in the current sample. Overall, 261 of 288 predictions were met and the correspondence index was .84 (*p* = .0004). These values are consistent with values in previous research (e.g., Dowgwillo et al., 2018) and suggest that the circumplex structure was adequate in this sample. The IIP-SC has been extensively validated using both college and clinical samples (e.g., Hopwood et al., 2008; McCloskey et al., 2021), and distinct interpersonal problem profiles have been identified for individual personality disorders using various assessment measures (Williams & Simms, 2016; Wilson et al., 2017).

#### Interpersonal Sensitivities Circumplex

The ISC (Hopwood et al., 2011) is a 64-item measure that maps social allergens (interpersonal behaviors enacted by others that the respondent finds bothersome) onto the eight octants of the IPC. Items are rated on an 8-point Likert-type scale ranging from 1 (*not at all, never bothers me*) to 8 (*extremely, always bothers me*). In the current study, internal consistencies of ISC octants were measured using McDonald’s ω_*t*_ and ranged from .74 (LM) to .88 (HI) with a mean ω_*t*_ of .82. In this sample, a randomization test of hypothesized order relations (RANDALL; Tracey, 1997) was employed to ensure that the ISC adhered to a circumplex structure. Overall, 242 of 288 predictions were met and the correspondence index was .69 (*p* = .0004). Prior research supports the circumplex structure and criterion validity of the ISC (Hopwood & Good, 2019; Williams et al., 2014).

### Validity Scale

The Personality Assessment Inventory Infrequency Scale (PAI-Infrequency; Morey, 1991) is an eight-item scale used as a protocol validity measure to assess respondent inattention and carelessness. Items are rated on a 4-point Likert-type scale ranging from 0 (*false, not at all true*) to 3 (*very true*).

### Procedure

The study was approved through the Institutional Review Board or Pennsylvania State University. Participants completed a brief demographic questionnaire, including sex, race, age, and whether English is the participant’s primary language, as well as all STPP, IPC, and validity measures online using a standardized web-based interface (Qualtrics). Participants received course credit for their participation.

### Data Analyses and Interpretation

Data analyses were computed using a two-step procedure. Both steps were conducted using the “circumplex” package (version 0.3.6) developed for the R statistical platform (Girard et al., 2020). Both steps were repeated for problems and sensitivities IPC surfaces, as distinct patterns of interpersonal associations may emerge across surfaces, providing a richer differentiation of subscales.

In the first step, the SSM (Gurtman, 1992; Gurtman & Pincus, 2003) was used to calculate the elevation, amplitude, angular displacement, and *R*^2^ parameters of MSS, SPQ, FFSI, and PID-5 STPD subscale scores. Bootstrapping methodology (Zimmermann & Wright, 2017) was used to compute CIs around elevation, amplitude, and angular displacement parameters to make inferential conclusions regarding SSM parameters. This step also helped establish the elevation, distinctiveness, theme and prototypicality of the subscale scores, and therefore tests H1 to H4. An elevation parameter below the cutoff indicates that the target construct is unrelated to the general factor of the particular IPC surface, such as interpersonal distress or hypersensitivity. An amplitude parameter below the cutoff indicates that the target construct is not distinctive in its interpersonal content beyond the elevation. An *R*^2^ value below the cut-off indicates a complex profile and that the target construct does not have a unified interpersonal theme.

In the second step, within-surface interpersonal profiles were statistically compared by creating CIs around the SSM parameter differences between each pair of subscales that were expected to converge (H5). Bootstrapped CIs around difference scores were used to empirically evaluate whether the interpersonal profiles of two subscales are statistically distinct or overlapping. If the SSM parameter CIs for subscale differences do not contain zero, this indicates that there are statistically significant differences between the interpersonal content of the two subscales that are being compared (Dowgwillo & Pincus, 2017; Zimmermann & Wright, 2017). In addition to statistical significance, we adopted a heuristic criterion of a magnitude of least 22.5° (half an octant) for difference scores on angular displacement to be interpreted as distinct, to provide more appropriate content interpretations. This conservative approach was used due to the high power in the study, as there is no evidence to suggest that two scales less than 22.5° apart on the IPC have meaningful differences in content. If a subscale did not meet the *R*^2^ or amplitude cut-offs, it was excluded from convergence and divergence analyses. Exploratory analyses of subscale differences examined whether the inclusion of social anxiety content impacts subscale convergences and divergences, by testing the differences between social anxiety subscales and other negative/interpersonal subscales that do not contain social anxiety content.

Online supplementary tables and figures include total score analyses. The data and the syntax for data analysis are available at https://osf.io/wzr2g/.

## Results

### Interpersonal Content

**Figure 3.**
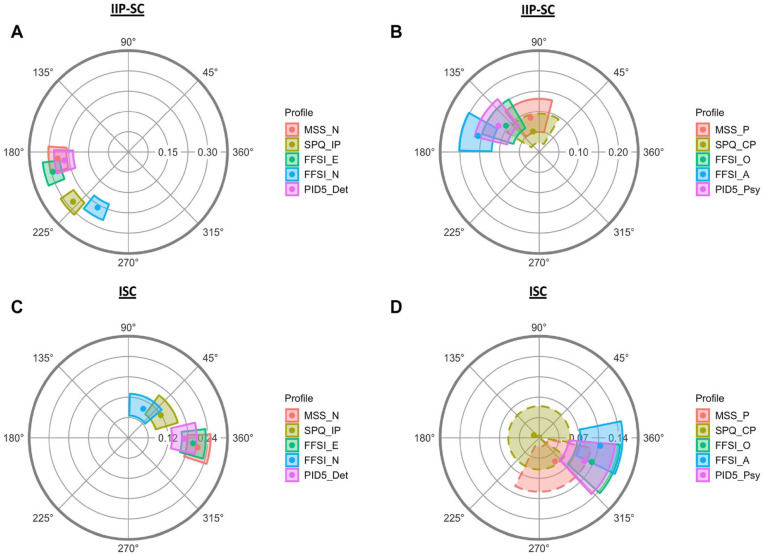
Amplitude and Angular Displacement CIs (SSM Plots) for Schizotypal Personality Pathology Subscales Across Interpersonal Problems (IIP-SC) and Sensitivities (ISC) Surfaces *Note.* Amplitude (radial) and angular displacement (angular) CIs for negative/interpersonal scales are depicted for negative/interpersonal schizotypy (A and C) and positive/cognitive-perceptual schizotypy (B and D) subscales and domains. Dashed lines indicate non-prototypical profiles (*R*^2^ < .70). SSM = structural summary method; IIP-SC = Inventory of Interpersonal Problems Short Circumplex; ISC = Interpersonal Sensitivities Circumplex; CI = confidence interval. Negative/interpersonal subscales: MSS_N = MSS Negative; SPQ_IP = SPQ Interpersonal; FFSI_E = FFSI Low Extraversion; FFSI_N = FFSI High Neuroticism; PID5_Det = PID-5 detachment; Positive/cognitive-perceptual subscales: MSS_P = MSS Positive; SPQ_CP = SPQ Cognitive-Perceptual; FFSI_A = FFSI Low Agreeableness; FFSI_O = FFSI High Openness; PID5_Psy = PID-5 psychoticism.

Tables 3 and 4 contain the structural summary parameters and the associated 95% bootstrapped CIs for all subscales on the problems and sensitivities surfaces. Visual representations of the CIs for the amplitude (radial CI) and the angular displacement (angular CI) for each surface are presented via circular SSM plots (Figure 3). This figure depicts the placement of STPP features assessed by subscales on the two IPC surfaces. Note that Figure 3 does not contain disorganized subscales or a visual representation of the CIs for elevation. The values reported in Tables 3 and 4 and Figure 3 were used to test subscale SSM parameter hypotheses (H1–H4).

**Table 3.** Correlation-Based IIP-SC Structural Summary Parameters and 95% Bootstrapped Confidence Intervals of Schizotypal Personality Pathology Subscales.

Scale	Communion	Agency	Elevation	Amplitude	Angle	R^2^
MSS Negative	−0.28[−0.32, −0.23]	−0.02[−0.07, 0.03]	**0.19**[0.15, 0.24]	**0.28**[0.24, 0.32]	184.2°[174.5°, 194.3°]	**.937**
MSS Positive	−0.03[−0.07, 0.02]	0.09[0.05, 0.13]	**0.17**[0.12, 0.22]	0.09[0.06, 0.14]	105.6°[75.0°, 131.9°]	**.937**
MSS Disorganized	−0.07[−0.11, −0.02]	0.02[−0.02, 0.06]	**0.31**[0.27, 0.36]	0.07[0.03, 0.11]	161.6°[127.1°, 201.1°]	**.727**
SPQ Interpersonal	−0.21[−0.24, −0.18]	−0.19[−0.22, −0.15]	**0.41**[0.37, 0.44]	**0.28**[0.25, 0.32]	221.9°[214.2°, 229.3°]	**.899**
SPQ Cog-perceptual	−0.02[−0.06, 0.03]	0.05[0.01, 0.09]	**0.34**[0.29, 0.38]	0.05[0.02, 0.09]	108.4°[51.2°, 160.2°]	.486
SPQ-BRU Interpersonal	−0.25[−0.29, −0.22]	−0.11[−0.15, −0.07]	**0.35**[0.31, 0.39]	**0.27**[0.24, 0.31]	203.8°[195.7°, 212.2°]	**.831**
SPQ-BRU Social Anxiety	−0.11[−0.14, −0.07]	−0.23[−0.27, −0.19]	**0.36**[0.32, 0.40]	**0.25**[0.22, 0.28]	245.1°[235.6°, 254.0°]	**.923**
SPQ Disorganized	−0.02[−0.06, 0.02]	0.04[−0.00, 0.08]	**0.33**[0.28, 0.37]	0.04[0.01, 0.09]	113.3°[40.6°, 172.2°]	**.767**
FFSI Agreeableness	−0.15[−0.19, −0.11]	0.04[−0.00, 0.08]	**0.30**[0.25, 0.34]	**0.15**[0.11, 0.20]	166.1°[151.2°, 181.9°]	**.769**
FFSI Extraversion	−0.29[−0.32, −0.25]	−0.08[−0.12, −0.03]	**0.30**[0.25, 0.34]	**0.30**[0.27, 0.33]	194.9°[186.7°, 203.1°]	**.916**
FFSI Neuroticism	−0.12[−0.15, −0.08]	−0.21[−0.25, −0.18]	**0.38**[0.34, 0.42]	**0.24**[0.21, 0.28]	240.8°[231.6°, 250.1°]	**.925**
FFSI Openness	−0.08[−0.13, −0.04]	0.06[0.02, 0.11]	**0.24**[0.20, 0.29]	**0.11**[0.06, 0.15]	143.5°[121.5°, 169.1°]	**.886**
PID-5 Detachment	−0.25[−0.29, −0.21]	−0.03[−0.07, 0.00]	**0.31**[0.27, 0.35]	**0.25**[0.21, 0.29]	187.7°[178.9°, 196.6°]	**.878**
PID-5 Psychoticism	−0.10[−0.14, −0.06]	0.07[0.02, 0.11]	**0.25**[0.20, 0.29]	**0.12**[0.08, 0.17]	147.4°[128.6°, 167.0°]	**.887**

*Note.* Numbers in brackets represent the 95% confidence interval for the associated structural summary parameter. Items in bold represent the SSM parameters that were above the established cut-offs, indicating that these parameters were interpretable. IIP-SC = Inventory of Interpersonal Problems Short Circumplex; MSS = Multidimensional Schizotypy Scale; SPQ = Schizotypal Personality Questionnaire; SPQ-BRU = Schizotypal Personality Questionnaire, Brief Revised (Updated); FFSI = Five-Factor Schizotypal Inventory; PID-5 = Personality Inventory for *DSM*-5; SSM = structural summary method.

**Table 4.** Correlation-Based ISC Structural Summary Parameters and 95% Bootstrapped Confidence Intervals of Schizotypal Personality Pathology Subscales.

Scale	Communion	Agency	Elevation	Amplitude	Angle	*R* ^2^
MSS Negative	0.21[0.17, 0.25]	−0.03[−0.08, 0.01]	−0.05[−0.11, 0.01]	**0.21**[0.18, 0.25]	351. 3°[340.8°, 2.9°]	**.892**
MSS Positive	0.03[−0.02, 0.08]	−0.04[−0.08, 0.00]	**0.12**[0.06, 0.19]	0.05[0.01, 0.10]	308.0°[226.0°, 0.3°]	.666
MSS Disorganized	0.06[0.02, 0.10]	−0.02[−0.05, 0.02]	0.08[0.02, 0.13]	0.06[0.03, 0.11]	345.2°[314.7°, 24.1°]	.655
SPQ Interpersonal	0.10[0.05, 0.14]	0.06[0.03, 0.10]	0.05[−0.02, 0.11]	**0.11**[0.08, 0.15]	33.2°[13.8°, 54.8°]	**.835**
SPQ Cog-perceptual	−0.01[−0.06, 0.03]	0.02[−0.02, 0.05]	**0.15**[0.09, 0.21]	0.02[0.01, 0.07]	129.5°[344.0°, 267.3°]	.306
SPQ Disorganized	0.03[−0.01, 0.07]	0.04[−0.00, 0.08]	0.06[−0.00, 0.13]	0.05[0.02, 0.09]	52.4°[358.4°, 108.4°]	.561
FFSI Agreeableness	0.11[0.07, 0.15]	−0.01[−0.05, 0.03]	**0.14**[0.08, 0.20]	**0.11**[0.07, 0.15]	355.4°[336.0°, 16.8°]	**.914**
FFSI Extraversion	0.20[0.16, 0.23]	−0.02[−0.06, 0.01]	−0.00[−0.06, 0.06]	**0.20**[0.16, 0.24]	353.0°[342.3°, 4.5°]	**.886**
FFSI Neuroticism	0.05[0.00, 0.09]	0.08[0.04, 0.12]	0.05[−0.01, 0.12]	0.09[0.06, 0.13]	59.9°[31.9°, 87.2°]	**.726**
FFSI Openness	0.10[0.06, 0.14]	−0.03[−0.07, 0.01]	0.06[−0.01, 0.12]	**0.10**[0.06, 0.15]	341.1[320.9°, 3.8°]	**.872**
PID-5 Detachment	0.17[0.13, 0.21]	−0.00[−0.04, 0.04]	0.06[0.00, 0.12]	**0.17**[0.13, 0.21]	359.9°[347.2°, 13.6°]	**.928**
PID-5 Psychoticism	0.08[0.04, 0.13]	−0.03[−0.07, 0.01]	0.09[0.03, 0.16]	0.09[0.05, 0.14]	340.7°[319.4°, 6.3°]	**.795**

*Note.* Numbers in brackets represent the 95% confidence interval for the associated structural summary parameter. Values in bold represent the SSM parameters that were above the established cut-offs, indicating that these parameters were interpretable. ISC = Interpersonal Sensitivities Circumplex; MSS = Multidimensional Schizotypy Scale; SPQ = Schizotypal Personality Questionnaire; FFSI = Five-Factor Schizotypal Inventory; PID-5 = Personality Inventory for *DSM*-5; SSM = structural summary method.

#### Negative/Interpersonal Subscales

The MSS Negative Schizotypy (MSS-N), SPQ Interpersonal (SPQ-IP), FFSI High Neuroticism (FFSI N+), FFSI Low Extraversion (FFSI E-), and PID-5 detachment subscales assess STPP features aligned with social, interpersonal and affective difficulties which result in detachment, anhedonia, flat affect and disinterest.

##### Interpersonal Problems

Table 3 contains the interpersonal problems SSM parameters for subscales and Figure 3A contains the SSM plot for negative/interpersonal subscales on the interpersonal problems surface. On the problems surface, *R*^2^ values for negative/interpersonal subscales indicate that all profiles were prototypical in nature, with all displaying a good fit to the curve. Amplitude values were above the established cut-off, indicating that all IIP-SC profiles were distinctive and all subscales are uniquely associated with particular interpersonal problems more so than others. Therefore, all angular displacement values were interpretable. Among the negative/interpersonal subscales, angular displacement values ranged from 184.2° (MSS-N) to 240.8° (FFSI N+). These values suggest that STPP features assessed by MSS-N, FFSI E-, and PID-5 detachment are associated with cold (DE) interpersonal problems, whereas STPP features assessed by SPQ-IP and FFSI N+ are associated with socially avoidant (FG) interpersonal problems.

##### Interpersonal Sensitivities

Table 4 contains the interpersonal sensitivities SSM parameters for subscales and Figure 3C contains the SSM plot for negative/interpersonal subscales on the interpersonal sensitivities surface. On the sensitivities surface, *R*^2^ values for negative/interpersonal subscales indicate that all profiles were prototypical in nature, with all displaying a good fit to the curve. Amplitude values for all subscales except FFSI N+ were above the established cutoff, suggesting that ISC profiles of MSS-N, SPQ-IP, FFSI E+, and PID-5 detachment were distinctive and these subscales are uniquely associated with particular social allergens (sensitivity to certain bothersome behaviors) more so than others. Therefore, the angular displacement values for all negative/interpersonal subscales except FFSI N+ were interpretable. Among these scales, interpretable angular displacement values ranged from 351.3° (MSS-N) to 33.2° (SPQ-IP). These values suggest that STPP features assessed by MSS-N, FFSI E-, and PID-5 detachment are associated with sensitivities to affection (LM), whereas STPP features assessed by SPQ-IP are associated with sensitivities to attention-seeking (NO).

#### Positive/Cognitive-Perceptual Subscales

MSS Positive Schizotypy (MSS-P) and SPQ Cognitive-Perceptual (SPQ-CP), FFSI Low Agreeableness (FFSI A-), FFSI High Openness (FFSI O+), and PID-5 psychoticism subscales assess STPP features aligned with unusual beliefs and perceptions, ideas of reference and persecution, and disruptions in behavior thought content including oddness and eccentricity.

##### Interpersonal Problems

Table 3 contains the interpersonal problems SSM parameters for subscales and Figure 3B contains the SSM plot for positive/cognitive-perceptual subscales on the interpersonal problems surface. On the problems surface, all *R*^2^ values except for that of SPQ-CP indicate that profiles were prototypical in nature and displayed a good fit to the curve. The amplitude and angular displacement values for SPQ-CP were not interpreted due to profile complexity. Of the interpretable amplitude values, all but that of MSS-P were above the established cut-off, indicating that the IIP-SC profiles for FFSI A-, FFSI O+ and PID-5 psychoticism were distinctive and scores on these subscales are uniquely associated with particular interpersonal problems more so than others. Therefore, the angular displacement values for these three subscales were interpretable. However, the angular displacement of MSS-P was not interpreted due to its undifferentiated interpersonal problems profile (low amplitude). Among the three interpretable positive/cognitive-perceptual subscales, angular displacement values ranged from 143.5° (FFSI O+) to 166.1° (FFSI A−). These values suggest that STPP features assessed by FFSI O+ and PID-5 psychoticism are associated with vindictive (BC) interpersonal problems, whereas FFSI A− is associated with cold (DE) interpersonal problems.

##### Interpersonal Sensitivities

Table 4 contains the interpersonal sensitivities SSM parameters for subscales, and Figure 3D contains the SSM plot for positive/cognitive-perceptual subscales on the interpersonal sensitivities surface. On the sensitivities surface, *R*^2^ values for all positive/cognitive-perceptual subscales except for MSS-P and SPQ-CP indicate that profiles were prototypical in nature and displayed an acceptable to good fit to the curve. The amplitude and angular displacement values for MSS-P and SPQ-CP were not interpreted due to profile complexity. Of the interpretable amplitude values, those of FFSI A− and FFSI O+ were above the established cut-off, suggesting that the ISC profiles for these two subscales were distinctive and these scales are uniquely associated with certain social allergens (sensitivity to certain bothersome behaviors) more so than others. The angular displacement of PID-5 psychoticism was not interpreted due to its undifferentiated interpersonal sensitivities profile. Angular displacement values for FFSI A− and FFSI O+ were 355.4° and 341.1°, respectively. These values suggest that STPP features assessed by FFSI A- and FFSI O+ are associated with sensitivities to affection (LM). The ISC elevation values suggest that profiles for subscales measuring suspiciousness and ideas of reference in content (MSS-P, SPQ-CP, and FFSI A−) displayed small but interpretable positive elevations in general interpersonal hypersensitivity.

#### Disorganized Subscales

Disorganized subscales consist of MSS Disorganized (MSS-D) and SPQ Disorganized (SPQ-D). Tables 3 and 4 contain the interpersonal problems SSM parameters for disorganized subscales, although these were excluded from the SSM plots due to the lack of a priori hypotheses. These parameter values were calculated for exploratory purposes. On the problems surface, both subscales had prototypical but nondistinctive profiles and with large elevations (.31, .33), indicating that although interpersonally undifferentiated, disorganized subscales are strongly associated with general interpersonal distress. On the sensitivities surface, neither subscale reached an acceptable fit to the curve or exceeded the cut-off for elevation.

#### Summary

Confirming H1, all subscale elevations on the problems surface were above the cutoff. Confirming H2, angular displacement values of all subscales on the sensitivities surface were interpersonally opposite to angular displacement values on the sensitivities surface. For the negative/interpersonal subscales, H3 was supported as MSS-N, FFSI E−, and PID-5 detachment were all associated with cold problems and SPQ-IP and FFSI N+ were associated with cold-submissive problems. For the positive/cognitive perceptual scales, H3 was partially supported as all subscales, except for FFSI O+ and PID-5 psychoticism on the problems surface, showed cold themes instead of cold-dominant themes. Confirming H4, MSS-P, SPQ-CP, and FFSI A−, all showed significant positive associations with general interpersonal hypersensitivity (ISC elevation).

### Difference Score Confidence Intervals

Tables 5 and 6 contain the differences among structural summary parameters associated with subscales and the 95% bootstrapped CIs for each difference score across the two IPC surfaces. These results were used to test subscale convergence and divergence hypotheses (H5). If a difference CI does not contain zero, this indicates that there are statistically significant differences between the interpersonal content of two subscales that are being compared on that SSM parameter. Due to convergence and divergence hypotheses being limited to angular displacement, differences in elevation and amplitude are not interpreted but are reported in Tables 5 and 6.

**Table 5.** Structural Summary Parameters With 95% CIs for Differences of Negative/Interpersonal Schizotypal Personality Pathology Subscales.

Scale	Communion	Agency	Elevation	Amplitude	Angle
IIP-SC					
MSS Negative vs. SPQ Interpersonal	**−0.05[−0.08, −0.01]**	**0.15[0.11, 0.19]**	**−0.22[−0.26, −0.17]**	−0.02[−0.06, 0.02]	***−32.9°[−43.1°, −23.8°]***
MSS Negative vs. FFSI Extraversion	0.02[−0.00, 0.05]	**0.04[0.01, 0.07]**	**−0.10[−0.13, −0.07]**	**−0.03[−0.06, −0.01]**	−7.2°[−14.0°, −0.5°]
MSS Negative vs. FFSI Neuroticism	**−0.14[−0.18, −0.10]**	**0.17[0.12, 0.22]**	**−0.19[−0.23, −0.14]**	0.02[−0.02, 0.07]	***−53.0°[−65.0°, −41.4°]***
MSS Negative vs. PID-5 Detachment	−0.02[−0.06, 0.01]	0.01[−0.03, 0.05]	**−0.11[−0.15, −0.07]**	0.02[−0.01, 0.05]	−2.2°[−10.7°, 6.3°]
SPQ Interpersonal vs. FFSI Extraversion	**0.07[0.05, 0.10]**	**−0.10[−0.13, −0.07]**	**0.13[0.10, 0.16]**	−0.02[−0.05, 0.01]	***25.4°[18.8°, 31.9°]***
SPQ Interpersonal vs. FFSI Neuroticism	**−0.09[−0.12, −0.07]**	0.02[−0.00, 0.05]	**0.03[0.01, 0.05]**	**0.04[0.02, 0.07]**	**−20.0°[−26.5°, −13.3°]**
SPQ Interpersonal vs. PID-5 Detachment	**0.04[0.01, 0.07]**	**−0.15[−0.19, −0.12]**	**0.10[0.07, 0.14]**	0.02[−0.01, 0.06]	***34.1°[25.6°, 43.1°]***
FFSI Extraversion vs. FFSI Neuroticism	**−0.17[−0.20, −0.14]**	**0.13[0.09, 0.16]**	**−0.10[−0.13, −0.07]**	**0.06[0.03, 0.10]**	***−45.4°[−54.7°, −36.4°]***
FFSI Extraversion vs. PID-5 Detachment	**−0.04[−0.06, −0.02]**	**−0.04[−0.07, −0.02]**	−0.02[−0.05, 0.01]	**0.05[0.02, 0.07]**	7.5°[−0.4°, 16.4°]
FFSI Neuroticism vs. PID-5 Detachment	**0.13[0.10, 0.17]**	**−0.18[−0.22, −0.13]**	**0.07[0.04, 0.11]**	−0.02[−0.06, 0.02]	***53.2°[42.3°, 65.0°]***
ISC					
MSS Negative vs. SPQ Interpersonal	**0.12[0.08, 0.16]**	**−0.09[−0.13, −0.05]**	**−0.08[−0.15, −0.02]**	**0.10[0.06, 0.14]**	***−41.2°[−62.6°, −24.5°]***
MSS Negative vs. FFSI Extraversion	0.02[−0.00, 0.05]	−0.01[−0.03, 0.02]	−0.04[−0.08, 0.00]	0.02[−0.00, 0.05]	−1.5°[−8.7°, 4.6°]
MSS Negative vs. FFSI Neuroticism	**0.17[0.13, 0.21]**	**−0.11[−0.15, −0.07]**	**−0.08[−0.16, −0.02]**	**0.12[0.08, 0.17]**	—
MSS Negative vs. PID-5 Detachment	**0.04[0.01, 0.07]**	−0.03[−0.06, 0.01]	**−0.10[−0.15, −0.05]**	**0.04[0.01, 0.07]**	−7.0°[−17.7°, 2.7°]
SPQ Interpersonal vs. FFSI Extraversion	**−0.10[−0.13, −0.07]**	**0.09[0.06, 0.12]**	**0.05[0.00, 0.10]**	**−0.08[−0.11, −0.05]**	***42.5°[26.8°, 63.6°]***
SPQ Interpersonal vs. FFSI Neuroticism	**0.05[0.03, 0.07]**	−0.02[−0.04, 0.01]	−0.00[−0.04, 0.04]	0.02[−0.01, 0.04]	—
SPQ Interpersonal vs. PID-5 Detachment	**−0.08[−0.11, −0.04]**	**0.08[0.05, 0.11]**	**−0.00[−0.05, 0.05]**	**−0.05[−0.09, −0.02]**	***38.8°[22.8°, 60.2°]***
FFSI Extraversion vs. FFSI Neuroticism	**0.15[0.12, 0.18]**	**−0.10[−0.13, −0.08]**	**−0.05[−0.10, −0.00]**	**0.10[0.06, 0.14]**	—
FFSI Extraversion vs. PID-5 Detachment	**0.02[0.00, 0.05]**	−0.01[−0.04, 0.01]	**−0.06[−0.10, −0.02]**	**0.03[0.00, 0.05]**	−3.2°[−10.8°, 4.4°]
FFSI Neuroticism vs. PID-5 Detachment	**−0.13[−0.16, −0.09]**	**0.09[0.06, 0.13]**	−0.00[−0.06, 0.05]	**−0.08[−0.12, −0.03]**	—

*Note.* Numbers in brackets represent the 95% CI for the associated structural summary parameter. Items in bold represent parameters on which the compared scales are significantly distinct (CIs do not contain 0) and italics represent scale angles that differ by more than 22.5°. CI = confidence interval; IIP-SC = Inventory of Interpersonal Problems Short Circumplex; MSS = Multidimensional Schizotypy Scale; SPQ = Schizotypal Personality Questionnaire; FFSI = Five-Factor Schizotypal Inventory; PID-5 = Personality Inventory for *DSM*-5; ISC = Interpersonal Sensitivities Circumplex; SSM = structural summary method.

**Table 6.** Structural Summary Parameters With 95% CIs for Differences of Positive/Cognitive-Perceptual Schizotypal Personality Pathology Subscales.

Scale	Communion	Agency	Elevation	Amplitude	Angle (°)
IIP-SC					
MSS Positive vs. SPQ Cog-percep.	−0.01[−0.04, 0.03]	0.03[−0.01, 0.06]	**−0.14[−0.18, −0.10]**	—	—
MSS Positive vs. FFSI Agreeableness	**0.13[0.09, 0.17]**	0.04[−0.00, 0.08]	**−0.10[−0.14, −0.06]**	**−0.08[−0.12, −0.03]**	—
MSS Positive vs. FFSI Openness	**0.06[0.02, 0.09]**	0.02[−0.02, 0.06]	**−0.05[−0.09, −0.01]**	−0.02[−0.06, 0.03]	—
MSS Positive vs. PID-5 Psychoticism	**0.08[0.05, 0.11]**	0.02[−0.01, 0.05]	**−0.06[−0.10, −0.02]**	−0.03[−0.07, 0.01]	—
SPQ Cog-percep. vs. FFSI Agreeabl.	**0.14[0.11, 0.18]**	0.01[−0.02, 0.04]	**0.04[0.01, 0.08]**	—	—
SPQ Cog-percep. vs. FFSI Openness	**0.06[0.02, 0.10]**	−0.00[−0.04, 0.04]	**0.09[0.05, 0.13]**	—	—
SPQ Cog-percep. vs. PID-5 Psychoticism	**0.08[0.05, 0.12]**	−0.01[−0.04, 0.03]	**0.09[0.05, 0.13]**	—	—
FFSI Agreeabl. vs. FFSI Openness	**−0.08[−0.12, −0.04]**	−0.01[−0.05, 0.03]	**0.05[0.01, 0.09]**	**0.07[0.03, 0.11]**	19.7°[−2.3°, 43.3°]
FFSI Agreeabl. vs. PID-5 Psychoticism	**−0.06[−0.09, −0.02]**	−0.01[−0.05, 0.03]	**0.05[0.01, 0.09]**	**0.05[0.01, 0.08]**	14.6°[−1.7°, 31.7°]
FFSI Openness vs. PID-5 Psychoticism	0.02[−0.01, 0.04]	0.01[−0.02, 0.03]	−0.00[−0.03, 0.03]	−0.01[−0.04, 0.02]	−8.3°[−24.8°, 6.6°]
ISC					
MSS Positive vs. SPQ Cog-percep.	**0.04[0.01, 0.07]**	**−0.04[−0.07, −0.01]**	−0.03[−0.07, 0.02]	—	—
MSS Positive vs. FFSI Agreeabl.	**−0.08[−0.12, −0.05]**	−0.02[−0.06, 0.01]	−0.01[−0.05, 0.04]	—	—
MSS Positive vs. FFSI Openness	**−0.06[−0.10, −0.02]**	0.00[−0.04, 0.04]	**0.09[0.04, 0.14]**	—	—
MSS Positive vs. PID-5 Psychoticism	**−0.05[−0.08, −0.03]**	−0.00[−0.03, 0.03]	0.04[−0.00, 0.09]	—	—
SPQ Cog-percep. vs. FFSI Agreeabl.	**−0.12[−0.16, −0.09]**	0.01[−0.02, 0.04]	0.02[−0.02, 0.07]	—	—
SPQ Cog-percep. vs. FFSI Openness	**−0.10[−0.13, −0.06]**	**0.04[0.01, 0.07]**	**0.11[0.06, 0.17]**	—	—
SPQ Cog-percep. vs. PID-5 Psychoticism	**−0.09[−0.12, −0.05]**	**0.04[0.01, 0.08]**	0.07[0.01, 0.12]	—	—
FFSI Agreeabl. vs. FFSI Openness	**0.03[0.00, 0.06]**	0.03[−0.01, 0.06]	**0.09[0.04, 0.14]**	0.02[−0.01, 0.05]	**16.9[0.8, 36.4]**
FFSI Agreeabl. vs. PID-5 Psychoticism	**0.03[0.00, 0.07]**	0.03[−0.00, 0.06]	0.04[−0.01, 0.09]	0.02[−0.01, 0.06]	—
FFSI Openness vs. PID-5 Psychoticism	0.01[−0.01, 0.04]	0.00[−0.02, 0.02]	**−0.04[−0.08, −0.00]**	0.01[−0.01, 0.04]	—

*Note.* Numbers in brackets represent the 95% CI for the associated structural summary parameter. Items in bold represent parameters on which the compared scales are significantly distinct (CIs do not contain 0) CI = confidence interval; IIP-SC = Inventory of Interpersonal Problems Short Circumplex; MSS = Multidimensional Schizotypy Scale; SPQ = Schizotypal Personality Questionnaire; FFSI = Five-Factor Schizotypal Inventory; PID-5 = Personality Inventory for *DSM*-5; ISC = Interpersonal Sensitivities Circumplex; SSM = structural summary method.

#### Negative/Interpersonal Subscales

Parameter differences and associated difference CIs for subscales assessing negative/interpersonal STPP features are reported in Table 5. Recall that two sets of negative/interpersonal subscales were hypothesized to overlap within each other across IPC surfaces (H5a-b): (1) SPQ-IP and FFSI N+, (2) MSS-N, FFSI E- and PID-5 detachment.

##### Interpersonal Problems

The differences between the angular displacement parameter values for MSS-N, FFSI E-, and PID-5 detachment were nonsignificant, indicating that these subscales are angularly convergent with each other and associated with overlapping interpersonal problems. However, as expected, SPQ-IP and FFSI N+ exhibited significantly different angular displacement parameter values and significantly lower agency values compared with the other subscales. These differences indicate that SPQ-IP and FFSI N+ are more strongly associated with socially avoidant problems compared to the other three scales. Notably, the angular displacement values for SPQ-IP and FFSI N+ were significantly different, as well, suggesting that they are associated with divergent interpersonal problems themes, with FFSI N+ curving toward nonassertive (HI) problems. However, due to this difference being less than 22.5°, a meaningful interpretation of differences in interpersonal problems themes cannot be made.

##### Interpersonal Sensitivities

FFSI N+ was not included in the angular displacement comparisons due to its undifferentiated sensitivities profile. On the sensitivities surface, the differences between the angular displacement parameter values for MSS-N, FFSI E-, and PID-5 detachment were nonsignificant, indicating that these subscales are associated with overlapping social allergens. However, SPQ-IP displayed a significantly different angular displacement parameter and a higher score on the agency dimension compared with these three subscales, indicating a stronger association with sensitivity to attention-seeking (i.e., dominant and warm behaviors).

##### Summary

Overall, consistent with H5a-b, MSS N, FFSI E-, and PID-5 detachment had convergent angular displacement values and were associated with overlapping interpersonal themes across both surfaces. Although located in the same octant, SPQ-IP and FFSI N+ had statistically divergent angular displacement, however, due to this difference being less than 22.5°, a meaningful interpretation of differences in interpersonal problems themes cannot be made. Therefore, H5 was supported.

#### Positive/Cognitive-Perceptual Subscales

Parameter differences and associated difference CIs for subscales assessing positive/cognitive-perceptual STPP features are reported in Table 6. The set of subscales hypothesized to overlap consisted of the SPQ-CP, MSS-P, FFSI A−, FFSI O+, and PID-5 psychoticism subscales (H5c).

##### Interpersonal Problems

SPQ-CP was excluded from all profile comparisons due to profile complexity (low *R*^2^) on the problems surface. Furthermore, MSS-P was excluded from angular displacement comparisons due to its undifferentiated problems profile (low amplitude). The differences between angular displacement values for FFSI A−, FFSI O+, and PID-5 psychoticism were nonsignificant, suggesting that these subscales were angularly convergent and are all associated with vindictive (BC) interpersonal problems.

##### Interpersonal Sensitivities

SPQ-CP and MSS-P were excluded from all profile comparisons due to profile complexity (low *R*^2^). PID-5 psychoticism was excluded from angular displacement comparisons due to its undifferentiated sensitivities profile (low amplitude). On the sensitivities surface, the difference between the angular displacement values for FFSI O+ and FFSI A− was significant, suggesting angular divergence and association with different interpersonal sensitivities themes. Although FFSI A− and FFSI-O+ had statistically divergent angular displacement, due to this difference being less than 22.5°, a meaningful interpretation of differences in interpersonal problems themes cannot be made.

##### Summary

Overall, a majority of the interpretable angular displacement difference scores for positive/cognitive-perceptual subscales were convergent and associated with overlapping interpersonal themes, partially confirming H5c. Interpersonal themes of FFSI O+ and PID-5 psychoticism converged across both surfaces. FFSI A− diverged on the sensitivities surface.

### SPQ-BRU Social Anxiety Parameters and Comparisons (Exploratory Analyses)

**Figure 4.**
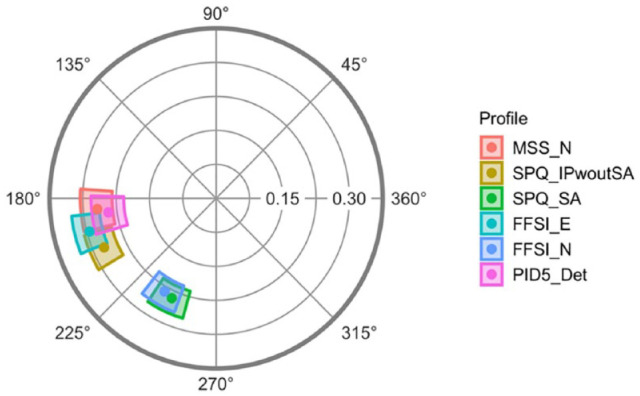
Amplitude (Radial) and Angular Displacement (Angular) Confidence Intervals for Negative/Interpersonal Schizotypal Personality Pathology Subscales Including SPQ-BRU Interpersonal and SPQ-BRU Social Anxiety on the IIP-SC Surface. *Note.* SPQ-BRU = Schizotypal Personality Questionnaire-Brief Revised Updated; IIP-SC = Inventory of Interpersonal Problems Short Circumplex; MSS_N = MSS Negative; SPQ_IPwoutSA = SPQ-BRU Interpersonal; SPQ_SA = SPQ-BRU Social Anxiety; FFSI_E = FFSI Low Extraversion; FFSI_N = FFSI High Neuroticism; PID5_Det = PID-5 detachment.

As noted earlier, SPQ and FFSI both include content related to social anxiety and nervousness in addition to social disinterest and anhedonia. Therefore, an exploration of the impact of social anxiety content on the interpersonal divergences of negative/interpersonal scales is warranted. Our exploratory analyses made use of the four-factor solution for the SPQ-BRU (Davidson et al., 2016) which separates the SPQ Interpersonal subscale into SPQ-BRU-IP (which taps social disinterest and anhedonia) and SPQ-BRU-SA (which taps social anxiety and nervousness). Analyses were only conducted with the IIP-SC as a representative IPC surface. IIP-SC structural summary parameters of SPQ-BRU-IP, SPQ-BRU-SA, and the other negative/interpersonal subscales are reported in Table 3. Parameter differences and associated difference CIs comparing SPQ-BRU-IP and SPQ-BRU-SA with each other and with the other negative/interpersonal subscales are reported in Table 7. Figure 4 shows the visual representations of the CIs for the amplitude (radial CI) and the angular displacement (angular CI) for SPQ-BRU-IP, SPQ-BRU-SA, and the other negative/interpersonal subscales.

**Table 7.** IIP-SC Structural Summary Parameters With 95% Bootstrapped CIs for Differences of SPQ-BRU Social Anxiety (SPQ-BRU-SA), SPQ Interpersonal Without Social Anxiety (SPQ-BRU-IP), and Other Negative/Interpersonal Schizotypal Personality Pathology Subscales.

Scale	Communion	Agency	Elevation	Amplitude	Angle
SPQ-BRU IP vs. SPQ-BRU SA	**−0.16[−0.20, −0.13]**	**0.10[0.06, 0.14]**	−0.01[−0.05, 0.02]	**0.05[0.01, 0.09]**	***−42.7°[−54.0°, −32.1°]***
FFSI Neuroticism vs. SPQ-BRU SA	−0.02[−0.04, 0.00]	0.01[−0.01, 0.03]	0.02[−0.00, 0.04]	0.00[−0.02, 0.02]	−4.9°[−11.2°, 0.9°]
FFSI Extraversion vs. SPQ-BRU SA	**−0.19[−0.22, −0.15]**	**0.13[0.10, 0.17]**	**−0.08[−0.12, −0.04]**	**0.06[0.03, 0.10]**	***−50.3°[−61.1°, −40.1°]***
MSS Negative vs. SPQ-BRU SA	**−0.16[−0.20, −0.12]**	**0.18[0.13, 0.23]**	**−0.17[−0.22, −0.12]**	0.03[−0.02, 0.07]	***−58.2°[−70.3°, −45.3°]***
PID5 Detachment vs. SPQ-BRU SA	**−0.15[−0.19, −0.11]**	**0.19[0.15, 0.23]**	**−0.05[−0.10, −0.01]**	0.01[−0.03, 0.06]	***−58.5°[−71.1°, −46.2°]***
FFSI Neuroticism vs. SPQ-BRU IP	**0.14[0.11, 0.17]**	**−0.10[−0.13, −0.06]**	0.03[−0.00, 0.06]	**−0.04[−0.08, −0.01]**	***37.5°[27.2°, 47.4°]***
FFSI Extraversion vs. SPQ-BRU IP	−0.03[−0.05, 0.00]	**0.03[0.00, 0.06]**	**−0.07[−0.10, −0.04]**	0.02[−0.01, 0.04]	**−7.9°[−14.4°, −1.7°]**
MSS Negative vs. SPQ-BRU IP	−0.00[−0.03, 0.04]	**0.07[0.03, 0.11]**	**−0.16[−0.20, −0.12]**	−0.02[−0.06, 0.02]	−**15.0°[−23.8°, −6.4°]**
PID-5 Detachment vs. SPQ-BRU IP	0.00[−0.03, 0.03]	**0.08[0.05, 0.11]**	**−0.05[−0.08, −0.02]**	−0.02[−0.06, 0.01]	**−16.2°[−23.9°, −9.0°]**

*Note.* Numbers in brackets represent the 95% CI for the associated structural summary parameter. Items in bold represent parameters on which the compared scales are significantly distinct (CIs do not contain 0) and italics represent scale angles that differ by more than 22.5°. IIP-SC = Inventory of Interpersonal Problems Short Circumplex; CI = confidence interval; SPQ-BRU = Schizotypal Personality Questionnaire-Brief Revised Updated; SA = Social Anxiety; IP = Interpersonal; FFSI = Five-Factor Schizotypal Inventory; MSS = Multidimensional Schizotypy Scale; PID-5 = Personality Inventory for *DSM*-5; SSM = structural summary method.

*R*^2^ values for both SPQ-BRU-IP and SPQ-BRU-SA subscales indicated that profiles were distinctive and prototypical. The angular displacement of SPQ-BRU Interpersonal was 203.8°. The angular displacement of SPQ Social Anxiety was 245.1°. These values indicate that SPQ-BRU-IP is associated with cold (DE) interpersonal problems, while SPQ-BRU-SA is associated with socially avoidant (FG) interpersonal problems. Bootstrapped difference tests confirm that angular displacement for SPQ-BRU-IP and SPQ-BRU-SA are significantly different.

The angular displacement differences were significant between SPQ-BRU-IP and all other negative/interpersonal subscales. However, all subscales except for FSSI Neuroticism were less than 22.5° apart from SPQ-BRU-IP. These findings suggest that SPQ-BRU-IP is associated with a similar interpersonal problems theme to all other subscales that tap social disinterest and anhedonia but not social anxiety. The differences between the angular displacement parameter values for SPQ-BRU-SA and all other negative/interpersonal subscales except for FFSI Neuroticism were significant and larger than 22.5°, suggesting that SPQ-BRU-SA has a convergent interpersonal problems theme with FFSI Neuroticism, and both subscales are significantly more strongly associated with socially avoidant problems.

## Discussion

This study formally examined the interpersonal content, similarities, and differences of four instruments assessing STPP within the interpersonal construct validation framework. The main objective of the study was to evaluate the interpersonal correlates and convergent validity of these four measures by deriving a detailed portrait of interpersonal dispositions associated with the underlying dimensions of STPP. To achieve this aim, we employed the SSM for circumplex data (Gurtman & Pincus, 2003) to generate SSM parameters and a bootstrapping methodology (Zimmermann & Wright, 2017) to create difference CIs that empirically test subscale convergences. This research expands upon the prior descriptive interpersonal investigations of STPP (e.g., Haslam et al., 2002; Williams & Simms, 2016; Wilson et al., 2017) by empirically testing differences in interpersonal content. Overall, a majority of the hypotheses were supported. Supplementing the subscale convergence and divergence findings, additional cross-subscale differences were computed as further exploration of construct validity based on the differences in social anxiety content.

### Interpersonal Content of STPP Subscales

To provide a rich portrait for the multidimensionality of STPP, the current study examined the interpersonal correlates of positive/cognitive-perceptual, negative/interpersonal, and disorganized subscales. All negative/interpersonal subscales showed differentiated profiles across surfaces, except for the ISC profile of FFSI N+. Overall, higher scores on subscales assessing social disinterest and anhedonia, isolation, detachment, and flattened affect (MSS-N, FFSI E- and PID-5 detachment) were most associated with cold interpersonal problems, consistent with previous interpersonal analyses of maladaptive detachment (Williams & Simms, 2016; Wright et al., 2012). Higher scores on subscales that assess social anxiety and discomfort (SPQ-IP and FFSI N+) compared with social disinterest (MSS-N, FFSI E−, and PID-5 detachment) were most associated with cold-submissive (i.e., socially avoidant) rather than purely cold interpersonal problems. These results indicate that subscales constructed to assess schizotypal social anxiety and discomfort interpersonally align with multiple measures designed to specifically assess social anxiety and FFM Neuroticism (Du et al., 2021; Erickson et al., 2016). Replicating previous research (Williams & Simms, 2016; Wright et al., 2012), STPP features assessed by FFSI O+ and PID-5 psychoticism exhibited modestly differentiated vindictive (cold-dominant) interpersonal problem themes. This provides further evidence for Wright and colleagues’ (2012) interpretation that psychoticism may show modest specificity in its association with vindictive interpersonal problems reflecting hostility toward other people due to discordant beliefs and realities.

In the current study, MSS-P and SPQ-CP had undifferentiated or complex profiles on multiple surfaces, although exhibiting modest to strong associations with general interpersonal distress and hypersensitivity. These results may be explained by the content of these positive/cognitive-perceptual subscales assessing psychotic-like expressions of schizotypy, such as supernatural experiences, unusual somatic experiences, and belief in possessing special powers. Thus, these symptoms may be associated with general rather than specific interpersonal correlates. Notably, consistent with hypotheses, positive/cognitive-perceptual subscales explicitly assessing suspiciousness and referential ideation (MSS-P, SPQ-CP, and FFSI-A+) were the only subscales in the current study that exhibited elevated interpersonal hypersensitivity. Previously, referential ideation and positive symptoms of schizophrenia have both been linked to making excessive mental state attributions to others (Fretland et al., 2015; Peyroux et al., 2019; Wastler & Lenzenweger, 2019). Taken together, these findings suggest that interpersonal hypersensitivity (i.e., the tendency to find others’ behaviors bothersome) may be related to hypermentalization (i.e., the tendency to make over-interpretive errors about others’ behaviors). This potential connection warrants a more explicit investigation in future studies.

### Convergences and Divergences of STPP Subscales

The current study examined the convergent validity of STPP subscales by hypothesizing that three sets of subscales will show associations with overlapping interpersonal themes: (a) subscales assessing social disinterest and anhedonia, (b) subscales assessing social anxiety, and (c) subscales assessing positive and cognitive-perceptual schizotypal personality features. Note that sets (a) and (b) were both included within negative/interpersonal subscale comparisons. Overall, convergence hypotheses about set (a), (b), and (c) were supported. Findings suggested that all set (a) subscales (i.e., MSS-N, PID-5, and FFSI E-) are associated with overlapping cold interpersonal problems and sensitivities to warmth and affection from others, establishing convergent validity. In addition, SPQ-IP and FFSI-N were associated with divergent interpersonal problems from all set (a) subscales and curved significantly more toward submission, providing strong evidence for discriminant validity. These findings therefore expand upon recent research showing that MSS-N and SPQ-IP do not exhibit strong associations with each other (Kwapil, Gross, Burgin, et al., 2018), suggesting that these two scales must be used cautiously, keeping in mind that they diverge in terms of interpersonal correlates. These findings further support that differences in social anxiety content contribute to the STPP subscale divergences. SPQ-IP and FFSI-N exhibited statistically distinct interpersonal problem themes (difference CIs did not contain 0), but their angular difference was less than 22.5°. Therefore, due to our heuristic rule for interpreting convergence, these two scales were interpreted as conceptually overlapping. However, the statistical significance of the difference CIs of negative/interpersonal subscales was identified as warranting further examination in the current study, prompting follow-up analyses of social anxiety discussed in the next section.

In terms of overlap of positive/cognitive-perceptual subscales, due to the undifferentiated profiles of MSS-P and SPQ-CP, a complete examination of set (c) angular displacement overlaps could not be conducted for these two scales to replicate previous research that established robust associations between positive schizotypy and STPD symptomatology (Kemp et al., 2021; Kwapil et al., 2022). However, notable patterns of overlap emerged between positive/cognitive perceptual subscales of FFSI and PID-5. Present findings indicate that FFSI O+ and PID-5 psychoticism are associated with overlapping interpersonal problem themes, providing strong evidence for the convergent validity of these subscales of maladaptive trait variants. Previous research on the alignment of PID-5 Psychoticism and FFM Openness has been inconclusive (Gore & Widiger, 2013; Griffin & Samuel, 2014; Watson et al., 2013; Wright & Simms, 2014). The inconsistencies in findings may be related to the differential overlap of FFM Openness with certain features of psychoticism and maladaptive openness. Previous studies have discussed that specific features of positive schizotypy (i.e., eccentricity, oddness, and fantasy proneness) more closely align with FFM Openness than other features like unusual beliefs and experiences (Crego & Widiger, 2016; Moorman & Samuel, 2018). Present construct validation was conducted at the domain level. However, to expand upon these findings using an interpersonal perspective, future work might provide finer-grained interpersonal construct validation by calculating SSM parameter differences at the facet level for FFM Openness, FFSI O+, and PID-5 psychoticism.

### Impact of Social Anxiety Content on the Assessment of STPP

To further explore whether the inclusion of social anxiety in the content of STPP measures impacts construct validity, we analyzed the divergences of negative/interpersonal subscales. The overlap between MSS Negative subscale and SPQ Interpersonal subscale has recently been called into question, with research failing to find strong associations between these two subscales. These studies proposed SPQ Interpersonal items tapping social anxiety, guardedness, and interpersonal discomfort as the potential explanation of this divergence (Gross et al., 2014, 2018; Kwapil, Gross, Burgin, et al., 2018). The findings of the current study provide empirical support for this interpretation. Exploratory analyses of angular convergence conducted using the four-factor SPQ-BRU indicated that partitioning social anxiety items out of SPQ Interpersonal impacts subscale convergences. In this study, SPQ-BRU-SA exhibited overlapping interpersonal content with FFSI Neuroticism and was associated with socially avoidant interpersonal problems. In addition, without social anxiety content, SPQ-BRU-IP was associated with cold interpersonal problems, consistent with all other subscales measuring social disinterest and anhedonia, including MSS Negative and FFSI Extraversion. This suggests that negative features of schizotypy assessed using the MSS have overlapping interpersonal correlates with detachment and social anhedonia assessed using SPQ and FFSI when social anxiety is excluded. This adds to previous research linking the multidimensional models of schizotypy with STPD symptomatology (Kemp et al., 2021).

Furthermore, SPQ-BRU-IP and SPQ-BRU-SA were associated with significantly divergent interpersonal themes, providing evidence of discriminant validity and supporting the four-factor SPQ structure proposed by Davidson and colleagues (2016). This finding is consistent with an earlier examination of the SPQ’s factor structure, which identified social anhedonia and social anxiety as separate factors, with social anxiety showing a positive association with neuroticism and a large negative association with extraversion (Chmielewski & Watson, 2008). Overall, using interpersonal construct validation, the present study finds that social anxiety is an interpersonally distinct construct from detachment and social anhedonia and demonstrates how differences in item content may impact the construct and convergent validity of scales that all purportedly assess STPP.

### Limitations

This study has several limitations. First, although a strength of the study was the large sample size, the use of a student sample may have limited the range of pathology represented in the sample and thus affected study results. Furthermore, this limitation coupled with the preliminary nature of the study necessitated assigning liberal cutoffs for the interpretation of SSM elevation and amplitude parameters. These liberal cutoffs have previously been employed in preliminary examinations of interpersonal content of personality pathology and self and interpersonal dysfunction and are therefore justified for the current study (Dowgwillo et al., 2018; Williams & Simms, 2016; Wu et al., 2022). Nevertheless, the results of this study must be interpreted with caution, and future research may warrant the use of more conservative cutoffs with more diverse and clinical samples. In addition, the use of a clinical sample presenting with schizotypal personality characteristics may more accurately tap the maladaptive features of this construct.

In a similar vein, the exclusive use of self-report measures may have also affected the results. Particularly when assessing constructs like psychoticism, positive schizotypy, and disorganized schizotypy, it is important to keep in mind that cognitive distortions, eccentricity, and unusual beliefs may lead one to unreliably report these as maladaptive and endorse items for features other than those they were constructed to assess (Kwapil, Gross, Silvia, et al., 2018). Previous work has reported correlations of .45 to .47 between MSS and interview-rated schizotypal symptoms (Kemp et al., 2021), which is comparable to correlations reported for self-other agreement for personality disorders (Klonsky et al., 2002). However, the use of informant ratings from close others or treating clinicians would advance this research. In fact, future work might address this by using the interpersonal construct validation method to specifically examine differences in structural summary parameters of self- and informant-ratings (e.g., self-report vs. interviews) of schizotypy and STPD. Despite these limitations, the current study is the first multisurface interpersonal examination of alternative STPP measures employing bootstrapped hypothesis tests, providing robust findings with multiple implications for convergent and discriminant validity.

## Conclusion

Expressions of negative schizotypal personality pathology, as assessed by subscales of four measures of interest in this study, are prototypically and distinctively interpersonal constructs associated with cold and socially avoidant interpersonal problems and hypersensitivity to others’ warmth and affection. Expressions of positive schizotypal personality pathology as assessed by subscales of FFSI and PID-5 are prototypically and distinctively interpersonal constructs associated with vindictiveness. Subscales assessing social disinterest and anhedonia converge with each other whereas subscales focusing on social anxiety are more strongly associated with avoidant interpersonal problems. Finally, FFSI High Openness and PID-5 psychoticism assess interpersonally overlapping constructs. This study identifies notable interpersonal convergences and divergences between the four STPP measures, providing novel implications for construct validity, and adds to the growing literature supporting interpersonal construct validation as a compelling method to substantively establish convergent and divergent validity of different scales constructed to assess similar external constructs (e.g., Dowgwillo et al., 2018; Dowgwillo & Pincus, 2017; Grove et al., 2019). Most notably, the findings of this study raise the conceptual question of whether social anxiety is a core feature of the construct of schizotypal personality pathology. Resolving this issue in future work would advance the assessment of this construct.

## Supplemental Material

sj-docx-1-asm-10.1177_10731911221143354 – Supplemental material for Examining Schizotypal Personality Scales Within and Across Interpersonal Circumplex Surfaces Click here for additional data file. Supplemental material, sj-docx-1-asm-10.1177_10731911221143354 for Examining Schizotypal Personality Scales Within and Across Interpersonal Circumplex Surfaces by A. Esin Asan and Aaron L. Pincus in Assessment
