# Supplementary material for: Examining Schizotypal Personality Scales Within and Across Interpersonal Circumplex Surfaces
Source: Assessment. 2023 Jan 11;30(7):2296–317. doi: 10.1177/10731911221143354 (PMC10478344; doi:10.1177/10731911221143354)
Supplement: sj-docx-1-asm-10.1177_10731911221143354 – Supplemental material for Examining Schizotypal Personality Scales Within and Across Interpersonal Circumplex Surfaces [file sj-docx-1-asm-10.1177_10731911221143354.docx]

**Figure S1**

*Amplitude (radial) and angular displacement (angular) confidence intervals (SSM plots) for Schizotypal Personality Pathology scale total scores across interpersonal problems (IIP-SC) and sensitivities (ISC) surfaces*

**
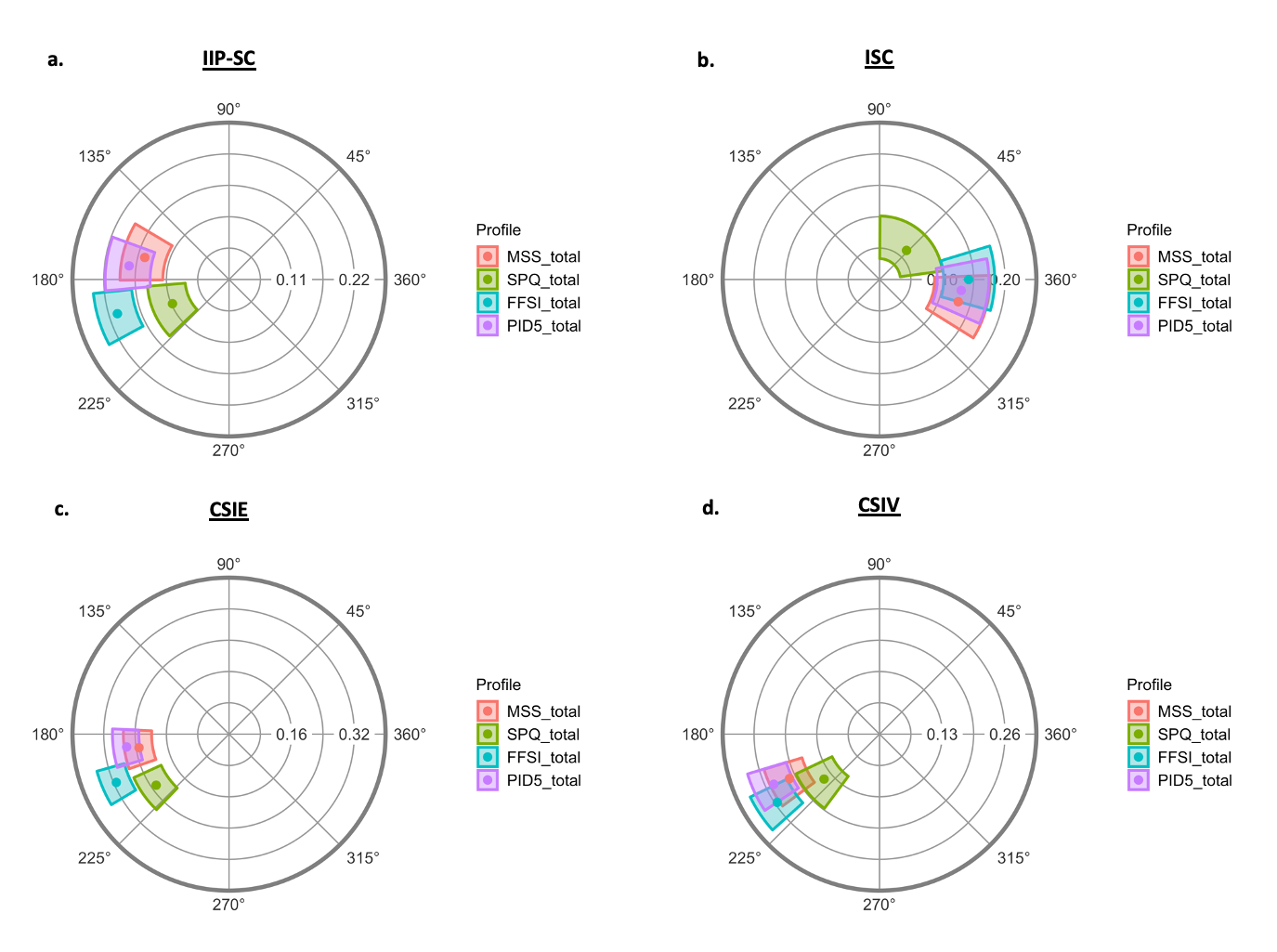
**

*Note.* MSS_total = Multidimensional Schizotypy Scale total score; SPQ_total = Schizotypal Personality Questionnaire total score; FFSI_total = Five-Factor Schizotyal Inventory total score;

PID-5_total = Personality Inventory for DSM-5 total score of Schizotypal Personality Disorder facets.

| **Table S1**  *Descriptive Statistics for Schizotypal Personality Pathology Scales* | | | | | |
| --- | --- | --- | --- | --- | --- |
| **Scale** | ***N*** | ***M*** | ***SD*** | **Min** | **Max** |
| MSS Total | 798 | 10.18 | 10.51 | 0.00 | 61.00 |
| SPQ Total | 855 | 2.47 | 0.62 | 1.00 | 4.34 |
| FFSI Total | 852 | 2.33 | 0.61 | 1.10 | 4.33 |
| PID-5 Total | 752 | 0.63 | 0.45 | 0.00 | 2.41 |

*Note.* Total *N* = 856; MSS = Multidimensional Schizotypy Scale; SPQ = Schizotypal Personality Questionnaire; FFSI = Five-Factor Schizotypy Inventory; PID-5 = Personality Inventory for DSM-5.

**Table S2**

*Correlations of total Schizotypal Personality Pathology scales and subscales with confidence intervals.*

| Scale | 1 | 2 | 3 | 4 | 5 | 6 | 7 | 8 | 9 | 10 | 11 | 12 | 13 | 14 | 15 |
| --- | --- | --- | --- | --- | --- | --- | --- | --- | --- | --- | --- | --- | --- | --- | --- |
|  |  |  |  |  |  |  |  |  |  |  |  |  |  |  |  |
| 1. MSS Total |  |  |  |  |  |  |  |  |  |  |  |  |  |  |  |
|  |  |  |  |  |  |  |  |  |  |  |  |  |  |  |  |
|  |  |  |  |  |  |  |  |  |  |  |  |  |  |  |  |
| 2. SPQ Total | .60** |  |  |  |  |  |  |  |  |  |  |  |  |  |  |
|  | [.56, .65] |  |  |  |  |  |  |  |  |  |  |  |  |  |  |
|  |  |  |  |  |  |  |  |  |  |  |  |  |  |  |  |
| 3. FFSI Total | .70** | .75** |  |  |  |  |  |  |  |  |  |  |  |  |  |
|  | [.66, .74] | [.72, .78] |  |  |  |  |  |  |  |  |  |  |  |  |  |
|  |  |  |  |  |  |  |  |  |  |  |  |  |  |  |  |
| 4. PID5 Total | .73** | .63** | .78** |  |  |  |  |  |  |  |  |  |  |  |  |
|  | [.69, .76] | [.58, .67] | [.75, .81] |  |  |  |  |  |  |  |  |  |  |  |  |
|  |  |  |  |  |  |  |  |  |  |  |  |  |  |  |  |
| 5. MSS Positive | .75** | .49** | .46** | .56** |  |  |  |  |  |  |  |  |  |  |  |
|  | [.72, .78] | [.44, .54] | [.41, .51] | [.51, .61] |  |  |  |  |  |  |  |  |  |  |  |
|  |  |  |  |  |  |  |  |  |  |  |  |  |  |  |  |
| 6. MSS Negative | .68** | .33** | .59** | .51** | .25** |  |  |  |  |  |  |  |  |  |  |
|  | [.64, .72] | [.26, .38] | [.54, .63] | [.45, .56] | [.18, .31] |  |  |  |  |  |  |  |  |  |  |
|  |  |  |  |  |  |  |  |  |  |  |  |  |  |  |  |
| 7. MSS Disorganized | .87** | .55** | .57** | .60** | .52** | .39** |  |  |  |  |  |  |  |  |  |
|  | [.85, .88] | [.51, .60] | [.52, .61] | [.55, .64] | [.47, .57] | [.33, .45] |  |  |  |  |  |  |  |  |  |
|  |  |  |  |  |  |  |  |  |  |  |  |  |  |  |  |
| 8. SPQ Cog. Perceptual | .56** | .86** | .57** | .54** | .62** | .18** | .48** |  |  |  |  |  |  |  |  |
|  | [.51, .60] | [.84, .87] | [.52, .61] | [.49, .59] | [.58, .66] | [.11, .24] | [.42, .53] |  |  |  |  |  |  |  |  |
|  |  |  |  |  |  |  |  |  |  |  |  |  |  |  |  |
| 9. SPQ Interpersonal | .45** | .78** | .70** | .49** | .19** | .45** | .39** | .46** |  |  |  |  |  |  |  |
|  | [.39, .50] | [.75, .81] | [.66, .73] | [.43, .54] | [.12, .25] | [.40, .50] | [.33, .45] | [.41, .51] |  |  |  |  |  |  |  |
|  |  |  |  |  |  |  |  |  |  |  |  |  |  |  |  |
| 10. SPQ Disorganized | .45** | .78** | .55** | .49** | .35** | .15** | .48** | .56** | .43** |  |  |  |  |  |  |
|  | [.39, .50] | [.76, .81] | [.50, .60] | [.43, .54] | [.29, .41] | [.08, .21] | [.43, .53] | [.51, .60] | [.37, .48] |  |  |  |  |  |  |
|  |  |  |  |  |  |  |  |  |  |  |  |  |  |  |  |
| 11. FFSI Openness | .61** | .60** | .80** | .74** | .54** | .34** | .52** | .52** | .33** | .64** |  |  |  |  |  |
|  | [.57, .66] | [.56, .64] | [.78, .83] | [.70, .77] | [.49, .59] | [.28, .40] | [.47, .57] | [.47, .57] | [.26, .38] | [.60, .68] |  |  |  |  |  |
|  |  |  |  |  |  |  |  |  |  |  |  |  |  |  |  |
| 12. FFSI Agreeableness | .62** | .65** | .78** | .67** | .50** | .44** | .51** | .62** | .52** | .39** | .60** |  |  |  |  |
|  | [.58, .66] | [.61, .68] | [.75, .81] | [.63, .71] | [.44, .54] | [.38, .49] | [.46, .56] | [.58, .66] | [.47, .57] | [.34, .45] | [.55, .64] |  |  |  |  |
|  |  |  |  |  |  |  |  |  |  |  |  |  |  |  |  |
| 13. FFSI Extraversion | .61** | .56** | .87** | .65** | .26** | .73** | .43** | .36** | .69** | .29** | .50** | .67** |  |  |  |
|  | [.56, .65] | [.51, .60] | [.85, .88] | [.60, .69] | [.20, .32] | [.70, .76] | [.38, .49] | [.30, .41] | [.66, .73] | [.23, .35] | [.44, .55] | [.63, .70] |  |  |  |
|  |  |  |  |  |  |  |  |  |  |  |  |  |  |  |  |
| 14. FFSI Neuroticism | .43** | .65** | .76** | .45** | .23** | .37** | .38** | .43** | .77** | .38** | .37** | .48** | .64** |  |  |
|  | [.37, .48] | [.61, .69] | [.73, .79] | [.39, .50] | [.16, .29] | [.31, .43] | [.32, .44] | [.38, .48] | [.74, .80] | [.32, .43] | [.31, .42] | [.42, .53] | [.60, .68] |  |  |
|  |  |  |  |  |  |  |  |  |  |  |  |  |  |  |  |
| 15. PID-5 Psychoticism | .69** | .56** | .67** | .94** | .63** | .35** | .58** | .55** | .32** | .50** | .77** | .57** | .46** | .32** |  |
|  | [.65, .72] | [.51, .61] | [.63, .71] | [.94, .95] | [.59, .67] | [.29, .41] | [.53, .63] | [.50, .60] | [.25, .38] | [.44, .55] | [.74, .79] | [.52, .62] | [.40, .51] | [.26, .38] |  |
|  |  |  |  |  |  |  |  |  |  |  |  |  |  |  |  |
| 16. PID-5 Detachment | .62** | .57** | .76** | .86** | .35** | .62** | .48** | .42** | .61** | .33** | .50** | .69** | .77** | .52** | .65** |
|  | [.58, .67] | [.52, .61] | [.72, .78] | [.84, .88] | [.29, .41] | [.57, .66] | [.43, .53] | [.36, .48] | [.57, .65] | [.26, .39] | [.45, .55] | [.65, .72] | [.75, .80] | [.47, .57] | [.61, .69] |
|  |  |  |  |  |  |  |  |  |  |  |  |  |  |  |  |

*Note.* Values in square brackets indicate the 95% confidence interval for each correlation. ** indicates *p* < .001; SPQ Cog. Perceptual = SPQ Cognitive-Perceptual.

| **Table S3**  *Correlation-based structural summary parameters and 95% bootstrapped confidence intervals for Schizotypal Personality Pathology scale total scores for interpersonal problems (IIP-SC) and sensitivities (ISC) surfaces* | | | | | | |
| --- | --- | --- | --- | --- | --- | --- |
| **IIP-SC** | | | | | | |
| **Scale** | **Communion** | **Agency** | **Elevation** | **Amplitude** | **Angle** | **R^2^** |
| MSS Total | -0.15  [-0.19, -0.11] | 0.04  [-00, 0.08] | **0.31**  [0.26, 0.35] | **0.16**  [0.12, 0.20] | 165.4°  [150.2°, 181.4°] | **0.922** |
| SPQ Total | -0.10  [-0.14, -0.07] | -0.04  [-0.08, -0.01] | **0.44**  [0.40, 0.48] | **0.11**  [0.08, 0.14] | 203.1°  [184.6°, 222.9°] | **0.771** |
| FFSI Total | -0.20  [-0.23, -0.17] | -0.06 [  -0.10, -0.02] | **0.37**  [0.33, 0.41] | **0.21**  [0.18, 0.24] | 197.0°  [186.5°, 208.0°] | **0.897** |
| PID5 Total | -0.18  [-0.22, -0.14] | 0.02  [-0.01, 0.07] | **0.30**  [0.26, 0.35] | **0.18**  [0.14, 0.22] | 172.2°  [159.2°, 184.9°] | **0.882** |
| **ISC** | | | | | | |
| **Scale** | **Communion** | **Agency** | **Elevation** | **Amplitude** | **Angle** | **R^2^** |
| MSS Total | 0.13  [0.08, 0.17] | -0.04  [-0.07, 0.00] | 0.07  [0.01, 0.13] | **0.13**  [0.09, 0.18] | 344.2°  [328.3°, 2.6°] | **0.827** |
| SPQ Total | 0.04  [0.00, 0.09] | 0.05  [0.01, 0.08] | **0.11**  [0.05, 0.18] | 0.06  [0.03, 0.10] | 47.1°  [11.2°, 88.8°] | **0.719** |
| FFSI Total | 0.14  [0.10, 0.18] | -0.00  [-0.04, 0.04] | 0.06  [-0.00, 0.12] | **0.14**  [0.10, 0.19] | 360.0°  [344.8°, 16.9°] | **0.855** |
| PID5 Total | 0.13  [0.09, 0.17] | -0.02  [-0.05, 0.02] | 0.08  [0.02, 0.15] | **0.13**  [0.09, 0.18] | 352.4°  [336.7°, 10.2°] | **0.878** |

*Note*. Numbers in brackets represent the 95% confidence interval for the associated structural summary parameter. Values in bold represent the SSM parameters that were above the established cut-offs, indicating that these parameters were interpretable. MSS = Multidimensional Schizotypy Scale; SPQ = Schizotypal Personality Questionnaire; FFSI = Five-Factor Schizotypal Inventory; PID-5 = Personality Inventory for DSM-5; IIP-SC = Inventory of Interpersonal Problems Short Circumplex; ISC = Interpersonal Sensitivities Circumplex.

| **Table S4**  *Structural summary parameters with 95% bootstrapped confidence intervals for differences of Schizotypal Personality Pathology scale total scores for interpersonal problems (IIP-SC) and sensitivities (ISC) surfaces* | | | | | |
| --- | --- | --- | --- | --- | --- |
| **IIP-SC** | | | | | |
| **Scale** | **Communion** | **Agency** | **Elevation** | **Amplitude** | **Angle** |
| MSS vs. SPQ | -0.04  [-0.07, -0.00] | **0.07**  **[0.03, 0.10]** | **-0.13**  **[-0.17, -0.10]** | 0.03  [-0.01, 0.06] | ***-33.6****°*  ***[-54.1°, -16.5°]*** |
| MSS vs. FFSI | **0.06**  **[0.03, 0.08]** | **0.08**  **[0.05, 0.12]** | **-0.06**  **[-0.09, -0.03]** | **-0.07**  **[-0.10, -0.04]** | ***-26.8°***  ***[-42.4°, -13.4°]*** |
| MSS vs. PID5 | 0.03  [-0.00, 0.06] | 0.01  [-0.02, 0.05] | 0.00  [-0.03, 0.04] | -0.02  [-0.06, 0.00] | -6.8°  [-18.4°, 4.2°] |
| SPQ vs. FFSI | **0.10**  **[0.07, 0.12]** | 0.02  [-0.01, 0.05] | **0.08**  **[0.05, 0.11]** | **-0.10**  **[-0.12, -0.07]** | 6.3°  [-6.7°, 20.3°] |
| SPQ vs. PID5 | **0.08**  **[0.05, 0.11]** | **-0.06**  **[-0.09, -0.03]** | **0.15**  **[0.11, 0.18]** | **-0.07**  **[-0.11, -0.04]** | ***27.2°***  ***[11.5°, 44.9°]*** |
| FFSI vs. PID5 | -0.02  [-0.04, 0.00] | **-0.08**  **[-0.11, -0.05]** | **0.07**  **[0.04, 0.10]** | 0.03  [0.00, 0.05] | ***22.9°***  ***[13.9°, 33.3°]*** |
| **ISC** | | | | | |
| **Scale** | **Communion** | **Agency** | **Elevation** | **Amplitude** | **Angle** |
| MSS vs. SPQ | **0.08**  **[0.05, 0.11]** | **-0.07**  **[-0.11, -0.04]** | -0.04  [-0.10, 0.01] | **0.06**  **[0.01, 0.10]** | -- |
| MSS vs. FFSI | -0.02  [-0.05, 0.01] | -0.03  [-0.06, 0.00] | 0.01  [-0.03, 0.06] | -0.02  [-0.04, 0.01] | **-11.7°**  **[-25.5°, -0.1°]** |
| MSS vs. PID5 | -0.00  [-0.03, 0.02] | -0.02  [-0.05, 0.01] | -0.01  [-0.06, 0.03] | -0.00  [-0.03, 0.03] | -8.4°  [-22.6°, 4.2°] |
| SPQ vs. FFSI | -0.10  [-0.12, -0.07] | **0.04**  **[0.02, 0.07]** | **0.07**  **[0.02, 0.11]** | **-0.08**  **[-0.11, -0.04]** | -- |
| SPQ vs. PID5 | -0.09  [-0.12, -0.06] | **0.07**  **[0.04, 0.10]** | 0.05  [-0.01, 0.10] | **-0.07**  **[-0.11, -0.03]** | -- |
| FFSI vs. PID5 | 0.01  [-0.01, 0.04] | **0.03**  **[0.00, 0.05]** | -0.03  [-0.07, 0.02] | 0.01  [-0.02, 0.03] | **11.7°**  **[1.5°, 23.0°]** |

*Note.* Values represent the degree to which the second scale listed differs from the first scale. Numbers in brackets represent the 95% confidence interval for the associated structural summary parameter differences. Items in bold represent parameters on which the compared scales are significantly distinct (CIs do not contain 0) and italics represent scale angles that differ by more than 22.5°.

MSS = Multidimensional Schizotypy Scale; SPQ = Schizotypal Personality Questionnaire; FFSI = Five-Factor Schizotyal Inventory; PID-5 = Personality Inventory for DSM-5; IIP-SC = Inventory of Interpersonal Problems Short Circumplex; ISC = Interpersonal Sensitivities Circumplex.

| **Table S5**  *IIP-SC structural summary parameters with 95% bootstrapped confidence intervals for differences of SPQ-BRU Social Anxiety (SPQ-BRU-SA), SPQ Interpersonal without Social Anxiety (SPQ-BRU-IP) and other negative/interpersonal subscales* | | | | | |
| --- | --- | --- | --- | --- | --- |
| **Scale** | **Communion** | **Agency** | **Elevation** | **Amplitude** | **Angle** |
| SPQ-BRU IP vs.  SPQ-BRU SA | **-0.16**  **[-0.20, -0.13]** | **0.10**  **[0.06, 0.14]** | -0.01  [-0.05, 0.02] | **0.05**  **[0.01, 0.09]** | ***-42.7°***  ***[-54.0°, -32.1°]*** |
| FFSI Neuroticism vs.  SPQ-BRU SA | -0.02  [-0.04, 0.00] | 0.01  [-0.01, 0.03] | 0.02  [-0.00, 0.04] | 0.00  [-0.02, 0.02] | -4.9°  [-11.2°, 0.9°] |
| FFSI Extraversion vs.  SPQ-BRU SA | **-0.19**  **[-0.22, -0.15]** | **0.13**  **[0.10, 0.17]** | **-0.08**  **[-0.12, -0.04]** | **0.06**  **[0.03, 0.10]** | ***-50.3°***  ***[-61.1°, -40.1°]*** |
| MSS Negative vs.  SPQ-BRU SA | **-0.16**  **[-0.20, -0.12]** | **0.18**  **[0.13, 0.23]** | **-0.17**  **[-0.22, -0.12]** | 0.03  [-0.02, 0.07] | ***-58.2°***  ***[-70.3°, -45.3°]*** |
| PID5 Detachment vs. SPQ-BRU SA | **-0.15**  **[-0.19, -0.11]** | **0.19**  **[0.15, 0.23]** | **-0.05**  **[-0.10, -0.01]** | 0.01  [-0.03, 0.06] | ***-58.5°***  ***[-71.1°, -46.2°]*** |
| FFSI Neuroticism vs.  SPQ-BRU IP | **0.14**  **[0.11, 0.17]** | **-0.10**  **[-0.13, -0.06]** | 0.03  [-0.00, 0.06] | **-0.04**  **[-0.08, -0.01]** | ***37.5°***  ***[27.2°, 47.4°]*** |
| FFSI Extraversion vs.  SPQ-BRU IP | -0.03  [-0.05, 0.00] | **0.03**  **[0.00, 0.06]** | **-0.07**  **[-0.10, -0.04]** | 0.02  [-0.01, 0.04] | **-7.9°**  **[-14.4°, -1.7°]** |
| MSS Negative vs.  SPQ-BRU IP | -0.00  [-0.03, 0.04] | **0.07**  **[0.03, 0.11]** | **-0.16**  **[-0.20, -0.12]** | -0.02  [-0.06, 0.02] | -**15.0°**  **[-23.8°, -6.4°]** |
| PID5 Detachment vs. SPQ-BRU IP | 0.00  [-0.03, 0.03] | **0.08**  **[0.05, 0.11]** | **-0.05**  **[-0.08, -0.02]** | -0.02  [-0.06, 0.01] | **-16.2°**  **[-23.9°, -9.0°]** |

*Note.* Values represent the degree to which the second scale listed differs from the first scale. Numbers in brackets represent the 95% confidence interval for the associated structural summary parameter differences. Items in bold represent parameters on which the compared scales are significantly distinct (CIs do not contain 0) and italics represent scale angles that differ by more than 22.5°.

MSS = Multidimensional Schizotypy Scale; SPQ-BRU = Schizotypal Personality Questionnaire – Brief Revised (Updated); FFSI = Five-Factor Schizotypal Inventory; PID-5 = Personality Inventory for DSM-5; IIP-SC = Inventory of Interpersonal Problems Short Circumplex; SA = Social Anxiety; IP = Interpersonal.
